# Exopolysaccharide Biosynthesis in *Rhizobium leguminosarum* bv. *trifolii* Requires a Complementary Function of Two Homologous Glycosyltransferases PssG and PssI

**DOI:** 10.3390/ijms24044248

**Published:** 2023-02-20

**Authors:** Kamil Żebracki, Aleksandra Horbowicz, Małgorzata Marczak, Anna Turska-Szewczuk, Piotr Koper, Klaudia Wójcik, Marceli Romańczuk, Magdalena Wójcik, Andrzej Mazur

**Affiliations:** Department of Genetics and Microbiology, Institute of Biological Sciences, Maria Curie-Skłodowska University, Akademicka 19 St., 20-033 Lublin, Poland

**Keywords:** exopolysaccharide, glycosyltransferase, *Rhizobium*–legume symbiosis, Wzx/Wzy-dependent pathway, operon, transcriptional unit

## Abstract

The Pss-I region of *Rhizobium leguminosarum* bv. *trifolii* TA1 comprises more than 20 genes coding for glycosyltransferases, modifying enzymes, and polymerization/export proteins, altogether determining the biosynthesis of symbiotically relevant exopolysaccharides. In this study, the role of homologous PssG and PssI glycosyltransferases in exopolysaccharide subunit synthesis were analyzed. It was shown that the glycosyltransferase-encoding genes of the Pss-I region were part of a single large transcriptional unit with potential downstream promoters activated in specific conditions. The Δ*pssG* and Δ*pssI* mutants produced significantly lower amounts of the exopolysaccharide, while the double deletion mutant Δ*pssI*Δ*pssG* produced no exopolysaccharide. Complementation of double mutation with individual genes restored exopolysaccharide synthesis, but only to the level similar to that observed for the single Δ*pssI* or Δ*pssG* mutants, indicating that PssG and PssI serve complementary functions in the process. PssG and PssI interacted with each other in vivo and in vitro. Moreover, PssI displayed an expanded in vivo interaction network comprising other GTs involved in subunit assembly and polymerization/export proteins. PssG and PssI proteins were shown to interact with the inner membrane through amphipathic helices at their C-termini, and PssG also required other proteins involved in exopolysaccharide synthesis to localize in the membrane protein fraction.

## 1. Introduction

Glycosyltransferases play crucial roles in the biogenesis of the bacterial cell envelope. Based on the characterized structures of known GTs, five structural classes have been distinguished: GT-A, GT-B, GT-C, GT-D, and GT-E [1]. In the three-dimensional structure of class A GTs, there are two tightly associated β/α/β Rossmann-like folds forming a β-sheet. These structures are characteristic of nucleotide-binding proteins. The active site of this class of enzymes is formed by the conserved Asp-X-Asp (DXD) motif, coordinating a divalent metal ion that neutralizes the phosphate group of the nucleotide sugar derivative and catalyzes protein conformation changes during catalysis. The DXD motif is contained within the N-terminal domain responsible for sugar-binding, while the C-terminal domain is responsible for the binding of the acceptor [2,3,4]. Proteins characterized by the GT-B fold have two β/α/β Rossmann-like domains localized opposite each other and connected by a short linker. The N- and C-terminal domains bind the donor and acceptor, respectively, and the donor binding is an event initiating a conformational change of the protein from open to closed, which facilitates the glycosylation reaction [2,5]. GT-C are integral hydrophobic membrane proteins with no Rossmann folds, but they are characterized by the presence of 8–13 transmembrane helices (TMH). The N-terminal catalytic domain is responsible for substrate binding, while the C-terminal domain with a globular structure has glycosyltransferase activity. The donors for this class of enzymes are sugar-phospholipid complexes, unlike GT-A and GT-B, which use nucleotide sugar derivatives [6,7,8]. The GT-D class of GTs is represented by the DUF1792 protein catalyzing the transfer of glucose from UDP-Glu to the O-linked hexasaccharide decorating *Streptococcus parasanuinis* Fap1 adhesin [9]. The GT-E group includes the *Staphylococcus aureus* N-acetyl-D-mannose transferase TagA, which transfers ManNAc from UDP-ManNAc to a glucosyldiphosphate bound to an undecaprenyl anchor to form C55-PP-GlcNAc-ManNAc [10].

*Rhizobium leguminosarum* bv. *trifolii* are soil bacteria able to induce nitrogen-fixing nodules on clover. Establishing an effective symbiosis is a multistage process; one of the key signals determining its effectiveness and specificity is the bacterial exopolysaccharide (EPS) [11,12,13]. EPS produced by *R. leguminosarum* bv. *trifolii* consists of glucose, glucuronic acid, and galactose in a 5:2:1 molar ratio, decorated by *O*-acetyl and pyruvyl substituents [14,15]. A characteristic feature of the biosynthetic pathway of such heteropolysaccharides, named the Wzx/Wzy-dependent pathway, is the synthesis of the complete repeating subunit in the cytoplasm in conjunction with a lipid anchor, usually undecaprenyl phosphate (UndPP), due to the activity of specific sugar-active enzymes—glycosyltransferases (GTs) [16,17]. Subunits are then flipped to the periplasmic leaflet of the inner membrane, polymerized, and the nascent polysaccharide chain is translocated to the cell surface [18].

The largest identified cluster of genes determining EPS biosynthesis in *R. leguminosarum* bv. *trifolii* TA1 (RtTA1) is the chromosomally located Pss-I region (~35 kbp). It comprises more than 20 genes coding, for e.g., GTs, enzymes modifying sugars in the basic EPS subunit with non-sugar decorations, and proteins responsible for the polymerization and secretion of EPS outside the cell [19,20]. Nine out of ten genes encoding GTs involved or assigned to the synthesis of the octasaccharide EPS subunit in RtTA1 are encoded within the Pss-I region. Among them, *pssS* and *pssCDE* genes, encoding GTs involved in the synthesis of the tetrasaccharide backbone chain [21,22,23,24], flank a group of genes encoding hypothetical acetyl- and pyruvyltransferases (*pssRM*, *pssK*), a membrane flippase (*pssL*), the activity of which results in exposure of the subunit on the periplasmic side of the inner membrane [25], and *pssJIHGF* genes encoding a galactosyltransferase PssJ [26], as well as hypothetical GTs postulated to be engaged in the biosynthesis of the side chain of the EPS subunit. The priming phosphoglycosyl transferase PssA is encoded beyond Pss-I [27,28]. Excluding *pssD* and *pssE* genes encoding ‘halves’ of one GT [24], there is still an excess of possibly involved glycosyltransferases relative to the number of hexose residues to be transferred in the side chain of the subunit, which remains unexplained. The Pss-I region is flanked by genes encoding proteins with a confirmed or predicted regulatory functions: *regA*, *mgl2,* and *pssV* located near the 5′ end of the Pss-I region, upstream of the *pssW* gene [29], and *pssZ* localized close to the 3′ end of the Pss-I [30].

The transcriptional organization of the Pss-I region has been partially described. Data on the transcriptional activity of genes involved in EPS biosynthesis in *R. leguminosarum* bv. *trifolii* Rt24.2 were gathered by measurement of reporter enzyme activity for plasmids carrying DNA fragments predicted in silico to serve promoter functions. The minimum promoter score (P) for the promoter function prediction was set at >0.7, and only these fragments were checked for transcriptional activity. The authors postulated that the region consisted of several monocistronic and several short polycistronic transcriptional units [21]. However, large operons related to the biosynthesis of polysaccharides have been demonstrated in bacteria, e.g., the *gum* operon (12 genes, ~15 kbp) responsible for the synthesis of xanthan gum in *Xanthomonas campestris* [31], the 18-kb large gene cluster essential for the biosynthesis of alginate in *Pseudomonas aeruginosa* [32], or the 11.65 kbp *exoHKLAMONP* gene cluster involved in the biosynthesis of EPS I in *Sinorhizobium meliloti* [33]. Determination of the transcriptional organization of a group of genes is important not only for understanding how they are expressed and regulated. It also significantly facilitates a more accurate genetic manipulation of the organism, e.g., the construction of non-polar mutations helping to define the functions of these genes.

In this work, the function of two hypothetical glucosyltransferases encoded by neighboring homologous genes *pssG* and *pssI* from the *pssI-pssH-pssG-pssF* cluster localized in the Pss-I region was studied to reveal the redundancy of GTs putatively engaged in the synthesis of the side chain of the EPS octasaccharide subunit. The transcriptional organization of genes encoding GTs responsible for EPS biosynthesis was determined with the use of detailed RT-PCR and transcriptional fusion approaches, allowing the construction of non-polar mutations in respective genes to define their functions. Single and double deletions of *pssI* and *pssG* were obtained, complemented, and analyzed with respect to their influence on EPS biosynthesis. The topological studies of PssI and PssG using the *phoAlacZα* dual reporter gene system, the subcellular localization analysis performed in *E. coli* and *Rhizobium* overexpressing respective recombinant proteins, and the in silico structural predictions provided evidence that PssG and PssI are cytoplasmic glycosyltransferases with a predicted structure typical for GT-A class, but strongly interacting with the membrane through amphipathic helices at their C-termini. Despite their overall sequence similarity, PssG and PssI turned out to be substantially different with respect to their interaction network with other GTs and with proteins of the EPS polymerization/export system as well as the impact of each gene on exopolysaccharide synthesis. The obtained data strongly suggest complementary rather than interchangeable roles of these two homologous proteins in the EPS biosynthesis of *R. leguminosarum* bv. *trifolii.*

## 2. Results

### 2.1. Genes Encoding Glycosyltransferases Involved in EPS Biosynthesis in R. leguminosarum bv. trifolii Are Part of a Single Transcriptional Unit

**Figure 1:**
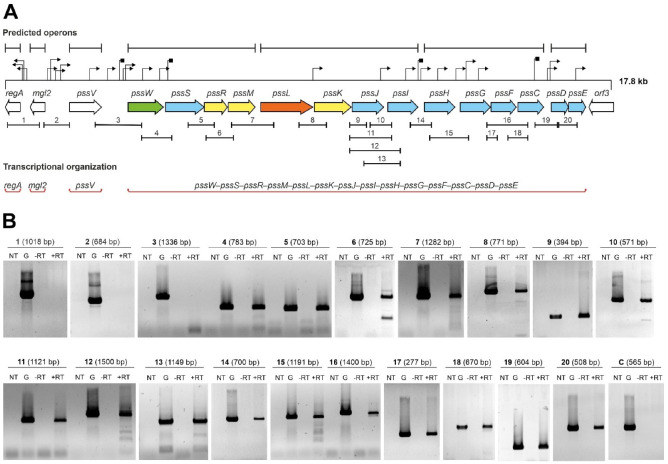
Analysis of the transcriptional organization of genes encoding GTs: (**A**) 17.8 kbp fragment of the 5′ end of the RtTA1 Pss-I region. The arrows represent individual open reading frames in the Pss-I region. The colors of the arrows indicate genes with the following confirmed or predicted functions: blue—GTs; yellow—EPS subunit modification; orange—flippase; green—EPS processing; and white—other or unknown functions. The upper part of the panel shows in silico predicted operons (black sections), promoters (black triangles), and terminators (black squares). The PCR-amplified fragments are marked below the arrows representing corresponding genes. The genes that have been experimentally verified to function as one operon are highlighted in red sections in the lower part of the panel. (**B**) Results of the PCR reactions with primers covering the intergenic regions of the studied gene cluster. The numbers correspond to the sections marked in panel (**A**). The sizes of the amplified DNA fragments are given in parentheses. The symbols mean: ‘NT’—control reaction without template DNA; ‘G’—control reaction with RtTA1 genomic DNA; ‘−RT’—control reaction with isolated total RNA without reverse transcription; and ‘+RT’—reaction with cDNA as a template. The amplicon marked as ‘C’ is an additional negative control to indicate no genomic DNA contamination of the RNA preparations (Supplementary Table S1).

In this study, we analyzed the transcriptional organization of the GT-encoding part of the Pss-I region in the RtTA1 strain (Figure 1A) by means of a detailed RT-PCR approach. The starting point was the in silico analysis of the examined fragment of the Pss-I region that was recently re-sequenced and annotated (GenBank Accession: MH595616, [26]). All genes encoding GTs in the *pssW*–*pssD* cluster have the same orientation and some reading frames partially overlap (*pssS* and *pssR*, *pssR* and *pssM*, *pssG* and *pssF*, *pssF* and *pssC*, *pssD* and *pssE*), which suggests common transcription for at least some of them. Computer predictions indicated the presence of many sequences that could act as promoters, which mapped particularly abundantly in the *pssJ*–*pssE* region and upstream of the *regA*, *pssV*, and *pssW* genes. Only motifs with the highest probability of functioning as promoters (both the value of the minimum promoter score *p* > 0.85 in Neural Network Promoter Prediction and the final score with a significant hit in PromoterHunter) were marked in Figure 1A. Hypothetical intrinsic Rho-independent terminator sequences were predicted downstream of the *pssV*, *pssW*, *pssI*, and *pssF* genes (Figure 1A). Two algorithms were used to predict operons in the studied region: one relies on gene conservation and genome architecture (Operon-mapper) and the other combines primary genomic sequence information with expression data from the RNA-seq data (Rockhopper). Transcriptomic data were obtained and published previously (BioProject Accession: PRJNA894372; [34]). In both cases, the tools split the 14 genes of the *pssW*–*pssE* cluster into 4 different transcriptional units, and genes upstream of this cluster were predicted to be expressed as individual monocistronic transcripts (Figure 1A).

To verify the transcriptional organization of the studied region experimentally, high-quality total RNA (free of genomic DNA) was isolated from RtTA1 cells according to a previously developed method [34]. cDNA was synthesized by reverse transcription with random primers and then used as a template in a series of PCR reactions with primers specific for pairs of adjacent genes (Supplementary Table S1). The observed profile of amplicons (Figure 1B) allowed us to conclude that the genes encoding the GTs form 1 operon (~13.5 kbp) composed of 14 genes, the transcription of which is initiated from the promoter located upstream of the *pssW* gene. The experimental data confirmed that the *regA*, *mgl2*, and *pssV* genes were not co-transcribed.

**Figure 2:**
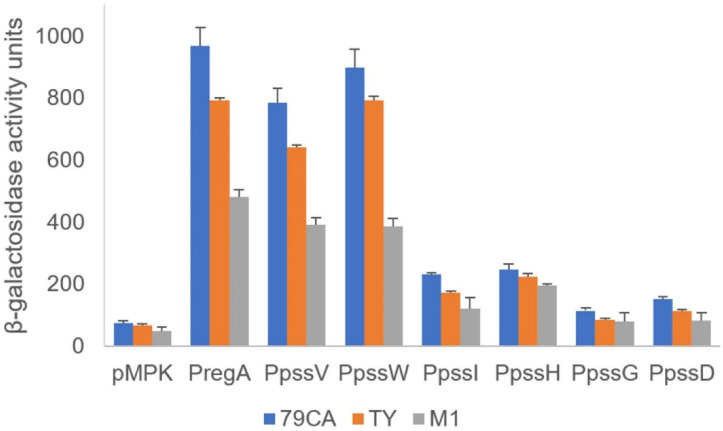
Transcriptional activity of hypothetical promoters in the studied fragment of the Pss-I region. PCR-amplified DNA fragments containing the predicted promoters upstream of the selected genes were cloned into the pMPK reporter vector. The β-galactosidase activities of the individual transcriptional fusions were expressed in Miller units. The presented results (with marked standard deviations) are the mean values of three independent experiments. The assays were carried out in complete (79CA and TY) and minimal (M1) media. All measured activities were significantly higher than the background (*p* < 0.01), except for the activities of the *pssG* and *pssD* promoters in M1 medium, which were insignificant.

The activity of the promoters located upstream of the *regA*, *pssV*, and *pssW* genes as well as the hypothetical ‘internal’ promoters upstream of selected GT genes in the operon were verified using transcriptional fusions with the *lacZ* reporter gene. Based on the promoter predictions, DNA fragments comprising the upstream regions of *regA*, *pssV*, *pssW*, *pssI*, *pssH*, *pssG*, and *pssD* were amplified and cloned upstream of a promoterless *lacZ* gene into the pMPK reporter vector. The activity of the *mgl2* gene promoter was studied previously [29]. The resulting plasmids (Supplementary Table S2) were introduced into the wild-type RtTA1 strain, and β-galactosidase activity was measured (Figure 2). The *regA*, *pssV*, and *pssW* genes were shown to be preceded by strong promoters (Figure 2), which was consistent with the RT-PCR results. The observed transcriptional activity pattern of these promoters was independent of the medium used for the growth of RtTA1.

The transcriptional activity of promoters upstream of *pssI*, *pssH*, *pssG*, and *pssD* were visibly lower, but still significantly above the background activity observed for the empty vector (Figure 2), especially for *pssI* and *pssH*. In the case of *pssG* and *pssD*, the activity of promoters were significantly higher than the background in 79CA and TY media, but insignificant in a case of M1 medium (Figure 2). The results obtained with the transcriptional fusion approach are not mutually exclusive with RT-PCR-based operon organization mapping; instead, they are complementary and show that the presence of a strong promoter in front of the operon does not exclude the existence of additional downstream promoters, which may be weak or inactive and can be activated in certain conditions.

### 2.2. PssG and PssI Proteins—Highly Similar, Yet Different

**Figure 3:**
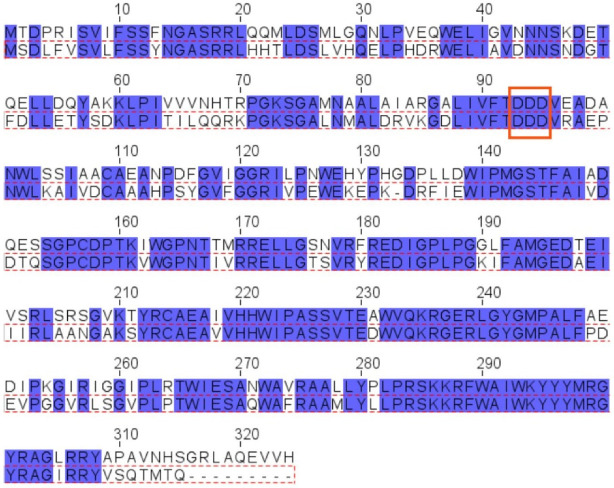
Alignment of amino acid sequences of PssG and PssI proteins. Alignment was performed with Clustal Omega and visualized in Jalview. Top aa sequence: PssG (QBN20832.1), bottom aa sequence: PssI (QBN20830.1) (red dotted frame). Blue boxes mark identical amino acids. Red frame marks the position of the DXD motif.

The *pssJ*-*pssI*-*pssH-pssG-pssF* genes encoding galactosyltransferase PssJ [26] and four hypothetical glucosyltransferases, probably involved in branching and elongation of the EPS subunit side chain, are clustered (Figure 1A). Two of these genes, *pssG* and *pssI*, show great similarity at the nucleotide level reaching 70% identity. The alignment of the amino acid sequences of both proteins revealed 68% identity/81% similarity. However, when the levels of similarity/identity in the N- and C-halves of both proteins were inspected separately, the N-terminal halves showed more dissimilarity than the C-terminal parts: N-halves (1–150 aa)—58% identity/74% similarity, C-halves (151–324 aa)—79% identity/90% similarity (Figure 3).

**Figure 4:**
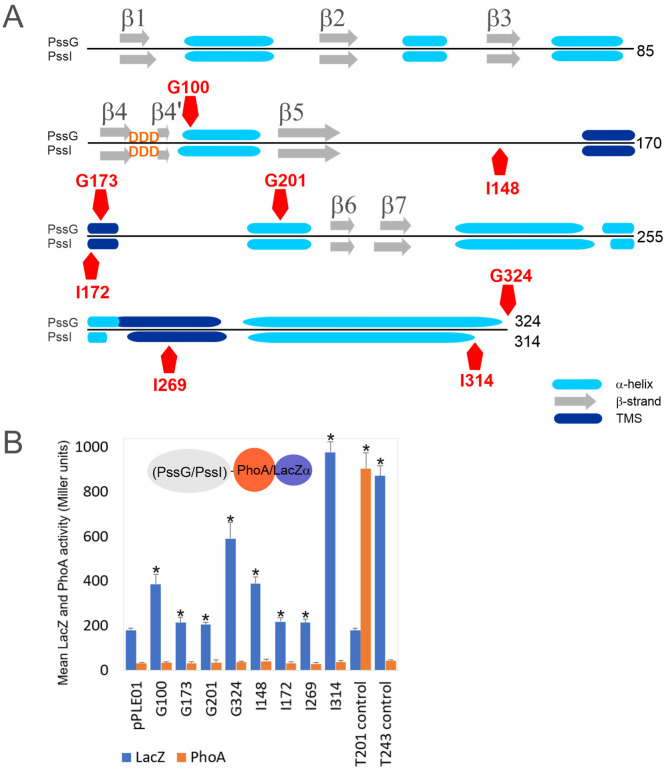
PssG and PssI proteins topology mapping using dual *phoAlacZα* reporter system. (**A**) The secondary structures scheme was based on Phyre2 prediction. Positions of fusion junctions were marked with red pentagons. (**B**) Graph presenting activity of reporter enzymes β-galactosidase (LacZ) and alkaline phosphatase (PhoA) measured in *E. coli* DH5α strain carrying appropriate fusion plasmids, along with the non-recombinant empty vector pPLE01. Bars represent the mean values of four independent experiments with two technical repeats each. T201 and T243 are control fusions with specific fragments of the *pssT* gene, with fusion junctions within periplasmic (T201) or cytoplasmic (T243) locations [34]. Asterisks (*) mark bars representing activities significantly higher than the background pPLE01 activity (*p* < 0.05).

The secondary structure prediction for both proteins revealed the presence of seven β-strands and several α-helices organized in a fashion typical for GT-A glycosyltransferases [2,35], where the N-terminal part folds into a sugar-nucleotide recognition domain with the characteristic DXD motif between two short β-strands, and the C-terminal domain is responsible for acceptor recognition (Figure 4A). If these proteins indeed act at the same stage of the octasaccharide synthesis, the similarity of C-terminal domains would suggest recognition of the same oligosaccharide-lipid anchor, while the dissimilarity of N-terminal domains would indicate differences in the specificity or strength of UDP-sugar binding. Despite the high level of their overall identity and similarity, both proteins are characterized by significantly different calculated pI, i.e., 8.38 for PssG and 7.06 for PssI, which may also reflect some functional divergence.

### 2.3. PssG and PssI Interact with the Inner Membrane through Amphipathic Helices

There are three topologically distinct classes of membrane proteins that glycosyltransferases may belong to: polytopic, bitopic, and monotopic. Current bioinformatics tools can relatively reliably predict transmembrane segments in poly- or bitopic proteins [36,37]. However, their effectiveness in prediction of topological determinants specific to monotopic proteins is limited [38].

We used CCTOP and Phyre2 tools to predict secondary structures and membrane topology, and to perform homology modeling of PssG and PssI. CCTOP predicted no transmembrane helices, while Phyre2 predicted the presence of two transmembrane helices in both protein sequences and proposed a model with N- and C-termini located in the cytoplasm and a loop between transmembrane segments (TMSs) exposed to the periplasm. Only minor differences in the length of the helices were observed in these proposed models. To verify whether PssG and PssI represent bitopic or monotopic membrane topology, we used a *phoAlacZα* dual reporter system, where PhoA and LacZ reporters were C-terminally fused to truncated or full-length versions of the *pssG* and *pssI* genes (Figure 4A,B). None of the fusions revealed any observable activity of alkaline phosphatase higher than the empty vector background, suggesting that none of the fusion junctions were translocated to the periplasm or localized within the membrane. Instead, all the fusions showed significantly higher activity of β-galactosidase than the control strain carrying the pPLE01 vector (Figure 4B). In fact, G100 and G324 (full-length PssG) as well as I148 and I314 (full-length PssI) revealed alkaline phosphatase/β-galactosidase activity ratios typical for cytoplasmic locations [39]. G173 and G201 (for PssG), as well as I172 and I269 (for PssI) with fusion junctions designed within the predicted α-helices or TMSs, showed very low activity of β-galactosidase, indicating stability/expression issues of respective fusion proteins (Figure 4B). Nevertheless, the activities were significantly higher than the background, supporting the non-periplasmic location of the fusion junctions.

**Figure 5:**
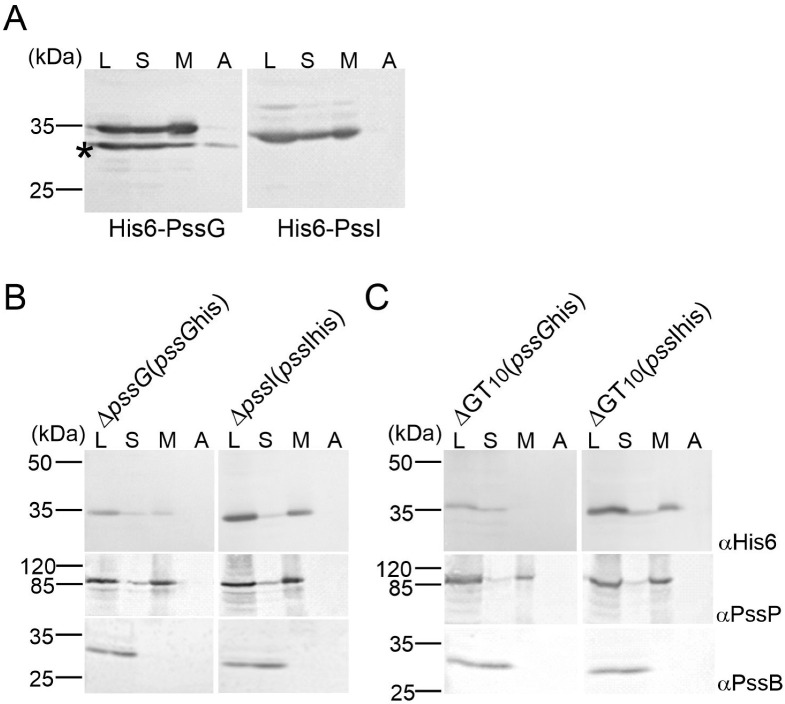
Study of the subcellular localization of PssG and PssI glycosyltransferases. Proteins of cell fractions obtained from *E. coli* M15 (pREP4) carrying pQE-30-based expression constructs (**A**), Δ*pssG* and Δ*pssI* mutants complemented with plasmids carrying *pssG* and *pssI* with a histidine-tag (**B**), and mutant ΔGT_10_ carrying the same complementation plasmids (**C**), were separated in SDS-PAGE and subjected to Western blotting with the following antibodies: anti-His_6_ (localization of tested proteins), anti-PssP (IM protein), and anti-PssB (cytoplasmic protein). The experiment was repeated twice with the same result. An asterisk (*) in (**A**) indicates protein species reactive with anti-His_6_ antibodies with an MW lower than calculated for PssG—probably shortened translation variant. L—cleared lysate; S—soluble proteins; M—membranes; A—membrane-associated proteins.

Topology mapping indicated the cytoplasmic location of PssG and PssI; however, since GTs act upon their substrates at the membrane interface, we additionally studied the subcellular localization of PssG and PssI by inspecting protein fractions obtained after centrifugation of two types of lysates*: E. coli* M15 (pREP4) carrying expression constructs in the pQE-30 vector and complemented versions of Δ*pssG* and Δ*pssI* single mutants (described below), where in trans introduced *pssG* and *pssI* ORFs were equipped with 6 histidine codons at the 3′-end (plasmids pBKpssG-His6 and pBKpssI-His6). Western blotting revealed that the PssG and PssI proteins were present in membrane fractions in both heterologous (Figure 5A) and homologous (Figure 5B) schemes and were not removed from the membrane by a high salt buffer. With the localization study and topology mapping results taken together, it can be proposed that PssG and PssI may either be monotopic GTs with α-helices embedded within a cytoplasmic leaflet of the inner membrane or interacting with the membrane through amphipathic helices at their C-termini. The result supporting the latter topology came from the localization study of recombinant His6-PssG in *E. coli*. An additional protein band with a molecular mass lower than expected—but possessing the tag—was observed in Western blotting for PssG (Figure 5A). Since the tag was localized at the N-terminus, the band must have represented a shorter translational variant of PssG. The protein distribution in the fractions was different from the full-length protein: there was visibly less protein in the membrane fraction, and some was even removed from the membrane with a high salt concentration (Figure 5A). The missing part may have represented an α-helix predicted at the C-terminus of the protein (Figure 4A).

Amphipathic helices are distinguished by their structure where hydrophobic and polar residues are segregated between two faces of the helix, which allows adsorbing at polar–apolar interfaces such as the membrane surface [40]. To verify the amphipathic character of the long α-helical secondary structures predicted in the C-termini of PssG and PssI (Figure 4A), ConSurf and HeliQuest tools were employed. ConSurf estimates the evolutionary conservation of amino acid positions in proteins based on the phylogenetic relations between homologous sequences—the degree of evolutionary conservation is dependent on the structural and functional importance of certain amino acids. HeliQuest examines whether a given segment of amino acids contains an uninterrupted ‘hydrophobic face’ of at least five amino acids that are adjacent when the sequence is represented on a helical wheel and if there is an analogous stretch of polar or poorly hydrophobic amino acids on the opposite side of the helical wheel.

**Figure 6:**
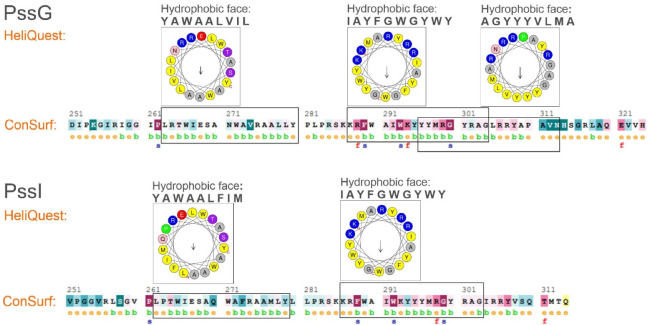
Verification of amphipathic character of C-terminal helices predicted in PssG and PssI proteins. Analyses were performed on C-terminal amino acid sequences, i.e., 251–324 aa for PssG, and 251–314 aa for PssI. HeliQuest: indicated parts of the amino acid sequences were analyzed to find segments, where the so-called ‘hydrophobic face’ could be identified in a helical wheel representation. Analyses were performed with parameters set at α-helices and an 18-aa window. The most representative results, i.e., the longest hydrophobic faces with poorly hydrophobic and polar residues on the opposite side of the helical wheel, were shown, and the corresponding amino acid sequences were framed. ConSurf: e—exposed residue, b—buried residues, f—functional residue (highly conserved and exposed), and s—structural residues (highly conserved and buried).

Our analyses confirmed that C-terminal 50–60 amino acids in both proteins form at least 2–3 stretches that may fold into amphipathic α-helices, with hydrophobic amino acids on one side and polar and charged amino acids on the opposite side. This specific organization represented on the helical wheels is nicely complemented by the ConSurf graphical results, where the amino acids in the predicted amphipathic stretches are organized in a typical pattern of 2–4 buried residues interwoven with 1 exposed residue (Figure 6).

### 2.4. PssG but Not PssI Localization to the Inner Membrane Requires Other Proteins Involved in EPS Synthesis

PssG and PssI were demonstrated to localize mainly to membrane fractions of lysed cells. This localization may be the result of not only specific structural features discussed above, but also protein–protein interactions between glycosyltransferases and other proteins involved in EPS synthesis. To test this assumption, protein fractionation was performed on the lysates of the ΔGT_10_ mutant, from which the *pssW*–*pssE* region and the *pssA* gene were removed from the chromosome using the *cre*-*loxP* technique, and then complementation plasmids pBKpssG-His6 or pBKpssI-His6 were introduced. These complemented strains produced only PssG-His_6_ or PssI-His_6_ from all the GTs involved in EPS synthesis. Inspection of fractions through Western blotting revealed that the location of the PssG protein changed in the genetic background of the ΔGT_10_ mutant and the protein was not detectable in the membrane fraction (Figure 5B). No such shift was observed for PssI, indicating that its presence in the membrane fraction does not depend on other glycosyltransferases involved in EPS synthesis (Figure 5B).

### 2.5. PssI Has a More Expanded BTH Interaction Network Than PssG

**Figure 7:**
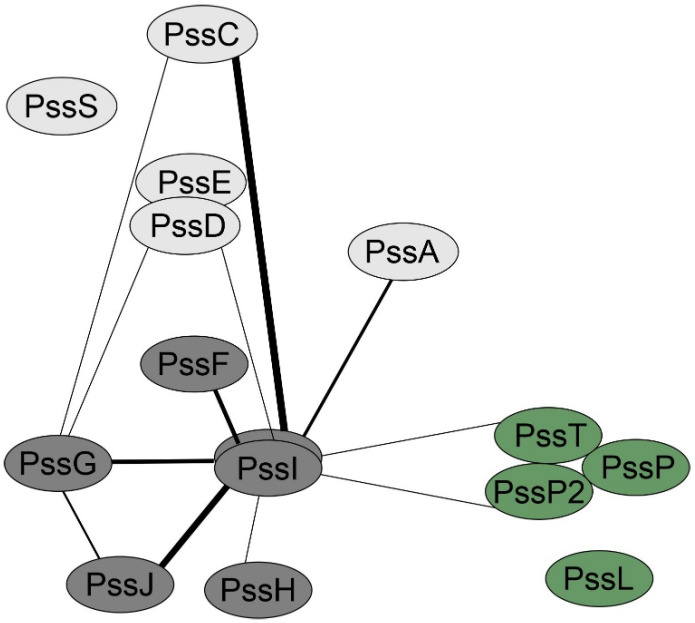
PssG and PssI interaction network created based on the bacterial two-hybrid screening results. The thickness of the lines connecting proteins is proportional to the number of pairs with significant activities from among all tested for any given protein pair. Activity values ± SD for all the tested fusion protein pairs were given in Supplementary Figure S1.

Considering the topology and localization study results, we tested whether PssG and PssI were indeed involved in an interaction network with other glycosyltransferases and components of the flipping/polymerization/export system of EPS. Screening for interactions in the bacterial two-hybrid system revealed that PssI was engaged in more interactions than PssG, once again suggesting their functional divergence (Figure 7). The most pronounced PssI partners, i.e., those for which interaction was observed in most combinations of PssI-other protein pairs (eight were possible), were glucuronosyltransferase PssC and GTs involved in side-chain assembly, with PssG among them (Figure 7). It is worth noting that PssI–PssT (polysaccharide polymerase) and PssI–PssP2 (secondary polysaccharide co-polymerase) interactions were also observed (Figure 7).

### 2.6. PssG and PssI Interact In Vitro

**Figure 8:**
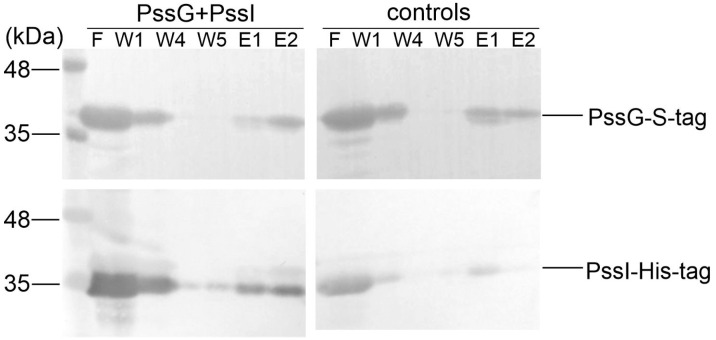
Pull-down verification of the PssG-PssI interaction revealed in a two-hybrid screening. PssG-S-tag (100 µg) and PssI-His6 (100 µg) proteins were mixed and applied to the S-tag affinity resin. After several rounds of washing (W1–W5), proteins were eluted from the resin and fraction composition was examined using Western blot with anti-His_6_ and anti-S-tag antibodies. Controls of the experiment were PssG-S-tag and PssI-His6, which were applied to the resin alone. F, flow; W, wash; E, elution. Top blots: detection with anti-S-tag antibodies; bottom blots: detection with anti-His_6_ antibodies.

We took advantage of the fact that recombinant GTs were easily packed into inclusion bodies and purified the recombinant PssG and PssI proteins equipped with S-tag and His_6_-tag, respectively, from the inclusion bodies; subsequently, they were subjected to refolding using the Thermo Scientific Pierce Protein Refolding Kit. Refolded proteins were then used in a pull-down assay to assess if they interacted in vitro and could be co-purified on affinity resin dedicated to S-tagged proteins. Analyses of protein fractions eluted from the resin revealed that PssI possessing the His_6_-tag binds to the column through the interaction with PssG, which is specifically bound to the resin through its S-tag at the C-terminus (Figure 8).

### 2.7. Single pssG or pssI Deletion Decreases the EPS Amount by Half

**Figure 9:**
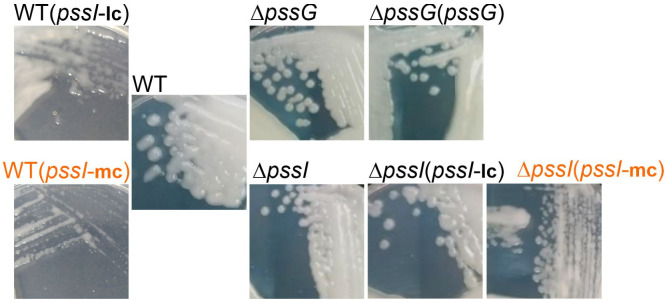
Macroscopic morphology of RtTA1 deletion mutants for the *pssG* and *pssI* genes, their derivatives after genetic complementation, as well as the wild-type strain carrying the same complementation plasmids. mc, medium copy number plasmid pBBRMCS-2 carrying *pssI* gene; lc, low copy number plasmid pRK7813 carrying *pssI* gene. Complementation of Δ*pssG* was performed only with pBBRMCS-2 derivative plasmid. Names in orange indicate strains where EPS production suppression was visible. All the photographs were taken 5 days post-inoculation.

Single gene deletions Δ*pssG* and Δ*pssI* were feasible to obtain using the *cre*-*loxP* system, but the frequency of detected homologous recombination events at the *pssG* and *pssI loci* was significantly different: 2.08% for *pssG* and 13.39% for *pssI*. The mutants produced EPS and no significant differences in the amount of EPS secreted in the agar medium were observed (Figure 9).

The complementation test for Δ*pssI* showed that the overexpression of the *pssI* gene resulting from its expression from P*lac* in the medium copy number plasmid pBBRMCS-2 resulted in inhibition of EPS biosynthesis (Figure 9). Normal EPS production was restored when the Δ*pssI* mutant was complemented with a construct based on a low copy number plasmid pRK7813 [Δ*pssI*(*pssI*-lc) strain]. Similar effects were observed when both these complementation plasmids were introduced into the wild-type RtTA1 cells: no suppression with the lc-plasmid and EPS production suppression with the mc-plasmid (Figure 9). Such a negative dominance effect is characteristic of multimeric proteins and indicates an important role of PssI in the network of proteins involved in EPS synthesis and/or transport. The observed effect confirms the relevance of the interactions observed in BTH screening.

**Figure 10:**
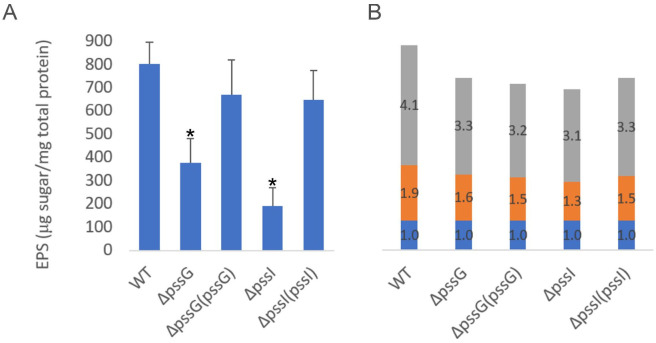
Analyses of exopolysaccharides produced by Δ*pssG*, Δ*pssI*, and their complemented derivatives. (**A**) The amount of total sugar in tested samples was determined based on the calibration curve prepared for the glucose solution and was expressed as the total sugar content (µg) per total protein of bacterial cells from the same culture sample (mg). Bars represent the mean values of four independent experiments with two technical repeats each. Error bars represent standard deviation. Asterisks (*) mark bars representing amounts significantly different than produced by the WT strain (*p* < 0.01). (**B**) Glycosyl composition analysis was performed by combined gas chromatography-mass spectrometry (GC/MS) of alditol acetates. Proportions of glucose (gray bars), glucuronic acid (orange bars), and galactose (blue bars) are shown. Bars represent the averaged duplicate results. In the case of the *pssI* gene, a complemented derivative Δ*pssI*(*pssI*-lc) was analyzed.

The quantitative analyses of exopolysaccharide secreted to the liquid medium revealed that the Δ*pssG* and Δ*pssI* mutants produced significantly lower EPS amounts (although it was not obvious after the inspection of growth in the agar medium), while complementation with *pssG* (cloned into pBBRMCS-2) and *pssI* (cloned into pRK7813) restored EPS production to nearly the wild-type level (Figure 10A). The glycosyl composition of exopolysaccharides produced by the mutants was not substantially different from the wild-type strain (Figure 10B). The Δ*pssG* and Δ*pssI* mutants showed slightly increased sensitivity to SDS and ethanol (Δ*pssG*) and increased resistance to deoxycholate (DOC) (Δ*pssI*) (Supplementary Figure S2). The latter finding, together with the slightly different effects of *pssG* or *pssI* mutations and overexpression on EPS production, further supports functional differences between these similar genes.

**Figure 11:**
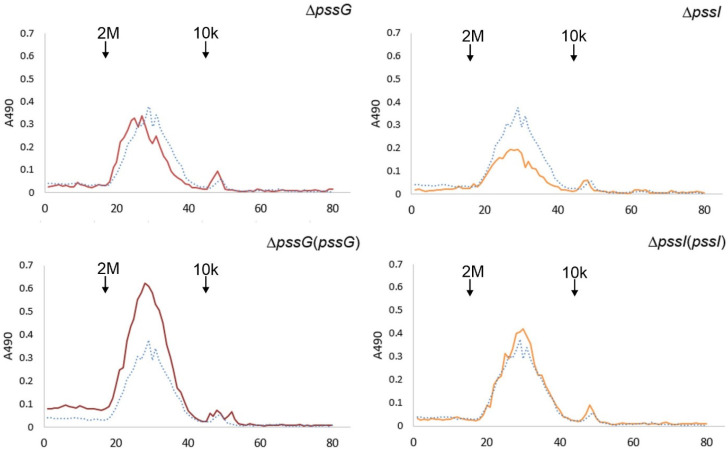
Gel permeation chromatography of exopolysaccharides produced by the wild-type strain RtTA1, mutants, and complementants for the *pssG* and *pssI* genes. Exopolysaccharides were precipitated with 95% ethanol from the culture supernatants. Dotted line—RtTA1, solid line—mutant or complementant indicated in the title. Molecular mass standards: 2 MDa, blue dextran, and 10 kDa, Dextran T10. In the case of the *pssI* gene, a complemented derivative Δ*pssI*(*pssI*-lc) was analyzed.

To verify whether the single deletions Δ*pssG* or Δ*pssI* had any consequences for the polymerization activity and the length of polysaccharide chains, gel permeation chromatography was performed. The profiles of the EPS samples of the mutants and complementants did not differ from the wild-type strain EPS profile in terms of specific mass distribution, indicating an undisturbed process of regulation of the polymerization degree (Figure 11). On the other hand, the efficiency of subunit flipping or polymerization may have been affected in Δ*pssG* and Δ*pssI* and contributed to the observed reduction in the amount of secreted EPS.

### 2.8. PssG and PssI Serve Complementary Functions in EPS Synthesis

**Figure 12:**
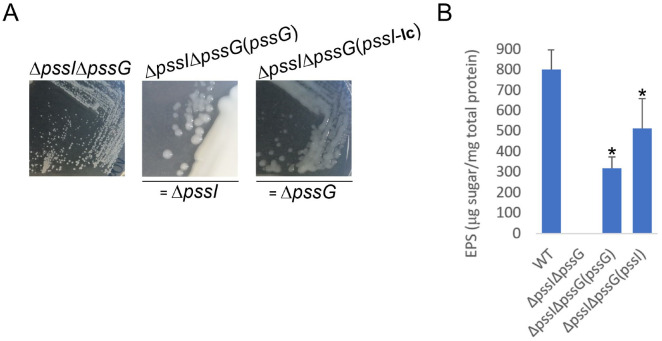
Macroscopic morphology of double deletion mutant Δ*pssG*Δ*pssI* and its partially complemented variants, with either *pssG* or *pssI* gene introduced. The same complementation plasmids, based either on pBBRMCS-2 or pRK7813, were used, as previously. In genetic terms, Δ*pssG*Δ*pssI*(*pssG*) corresponds to Δ*pssI* and Δ*pssG*Δ*pssI*(*pssI*) corresponds to Δ*pssG* (**A**). The result of EPS quantification in liquid culture supernatants is shown in (**B**). Bars represent the mean values of four independent experiments with two technical repeats each. Error bars represent standard deviation. Asterisks (*) mark bars representing amounts significantly different than produced by the WT strain (*p* < 0.01). The photographs in (**A**) were taken 5 days post-inoculation.

Considering the similarity between the PssG and PssI proteins, their interaction in vivo and in vitro, and the similar effect of single gene deletions in the reduction of the level of produced EPS at least by half, a complementary function of PssG and PssI was expected. Consequently, a double deletion Δ*pssI*Δ*pssG* mutant was constructed. The mutant did not produce any exopolysaccharide (Figure 12A). To establish whether the observed EPS-null phenotype resulted from the double deletion or selection of some other secondary mutation(s) in another locus/other loci, a complementation analysis was performed, where single *pssG* or *pssI* genes were introduced in trans to double mutant cells. Given the negative effects of *pssI* overexpression observed previously, we used a complementation construct based on pRK7813 for *pssI*. No such effect was observed for *pssG*, thus a medium copy number plasmid derivative was used for genetic complementation with this gene. The introduction of single genes restored EPS synthesis, but only to levels similar to those observed for the single Δ*pssI* and Δ*pssG* mutants (Figure 12B vs. Figure 10A), confirming that the lack of EPS production in the double mutant was specifically dependent on the concomitant lack of these two genes, and the level of EPS production suppression was specific and different for *pssG* and *pssI* alone.

## 3. Discussion

Genes involved in EPS synthesis in *R. leguminosarum* and *R. etli* are clustered in the chromosomal Pss-I region showing a high level of synteny between biovars and species [19,41,42]. The computationally deduced genetic organization of the region, i.e., partial overlapping of open reading frames and small intergenic regions, especially in the Pss-I segment encoding glycosyltransferases and EPS modification/processing enzymes, suggested their common transcription and regulation, likewise in other bacteria [43]. Janczarek et al. [21] postulated that genes in the Pss-I region of *R. leguminosarum* bv. *trifolii* Rt24.2 formed several monocistronic and several short polycistronic transcriptional units. However, the experimental scheme implemented in their work did not preclude other types of transcription organization.

We have evidenced that genes from the *pssW*–*pssE* cluster formed one transcriptional unit transcribed from the strong promoter present upstream of the *pssW* gene. However, the presence of additional weaker promoters in front of *pssI*, *pssH*, *pssG*, and *pssD* were also confirmed. The latter stays in agreement with data published by Janczarek et al. [21], but also with a present definition of an operon. The increasing number of transcriptomic data indicates that different subsets of genes in an operon may be co-transcribed in different conditions. A computational study of *E. coli* K12 transcriptomes [44] proved that different transcriptional units may overlap and share genes, and the terminators at the end of a cluster of functionally related genes are usually Rho-independent. The Pss-I region may be mainly transcribed as a single operon and considered as TUC (transcriptional units cluster) consisting of several smaller overlapping operons (sub-operons) [44]. In the analysis of the previously obtained RNA-Seq data [34], we noted that genes from the *pssW*–*pssE* transcriptional unit did not possess a uniform expression and had peaks of higher abundance of reads (TPM) mapping to the reference sequence (Supplementary Figure S4). In the case of a single polycistronic transcript, one should expect higher expression of the 5′ end than the 3′ end due to RNA polymerase processivity. The variations in the expression of co-transcribed genes suggest the existence of post-transcriptional mechanisms regulating the abundance of each transcript, such as the degradation of part of an mRNA or internal promoters within TUCs. It may also be due to the instability and degradation of long mRNA transcripts during the RNA isolation procedure. It should be noted that operon maps cannot be generalized for all growing conditions—they are rather specific for the conditions under which the transcriptome was analyzed. This phenomenon has been suggested for instance in *Helicobacter pylori* and *Mycobacterium tuberculosis* RNA-Seq studies, which demonstrated a wide prevalence of alternative transcriptional start sites within operons [45,46]. Additionally, Pelly et al. [47] assessed differences in operon arrangements in exponential and stationary growth phases of *M. tuberculosis* and found complex transcriptional regulation of transcriptional units occurring during specific growth phases.

It was interesting to note the abundance of potential and revealed promoters in the *pssI*-*pssH*-*pssG* genes cluster. It suggested that the expression of these genes could be differentially regulated in specific conditions. The activity of transcriptional fusion with P*pssI* was lower than in the case of P*pssH*, but it was similar to that of P*pssG*. Given the presence of the predicted Rho-independent terminator downstream the *pssI* gene, it seems valid that all three genes may belong to one TUC, but to different transcriptional units, according to the definitions introduced by Mao et al. [44]. Thus, the *pssI* and *pssG* genes encoding very similar glycosyltransferases may be regulated differently in yet unrecognized conditions.

This high similarity of genes and their predicted proteins products led to a hypothesis that the functions of PssG and PssI in the biosynthetic pathway of EPS may be similar or, given the sizes and plasticity of rhizobial genomes [48,49], identical and redundant. Both mutants deleted for single *pssG* or *pssI* genes produced less EPS with an undisturbed degree of polymerization and containing galactose. Considering the proposed activity of PssG and PssI as glucosyltransferases involved in transferring glucose residues to the EPS subunit side chain prior to the addition of terminal galactose, it remains to be answered whether such a phenomenon was caused by the broad specificity of terminal PssJ galactosyltransferase using shorter side chains as acceptor molecules for the galactose to be transferred or the fact that, due to the complementary activities of PssG and PssI, the side chains in EPSs of single mutants contain the same number of glucose residues as the EPS of the wild type strain.

If glucosyltransferase activities represented by PssG and PssI were redundant, the phenotype of single-gene mutants would be reminiscent of the dominant phenotype in the case of a heterozygous genotype in a 2n organism, where the remaining functional allele takes over the whole indispensable activity. Thus, in our model, the single *pssI* or *pssG* deletions should not have affected the amount of produced EPS. We have evidenced exactly the opposite effect. Considering this, we suspected that PssG and PssI could form a heterocomplex of two structurally similar proteins catalyzing the same step in the EPS subunit synthesis. Indeed, besides interacting in vivo when expressed in *E. coli*, the PssG and PssI proteins were also shown to interact in vitro when purified and refolded from inclusion bodies.

The *pssG* and *pssI* genes probably originated from a duplication event and can be recognized as paralogues. Gene duplication is frequently considered an important prerequisite for functional innovation facilitating adaptation to changing environments. Paralogous genes constitute a significant fraction of the bacterial genome coding capacity, and their number is correlated with the size of genomes. Duplicated genes in bacteria appear mainly via small-scale duplication events, and operons, relatively unstable throughout evolution, are prone to such events [50]. The large sizes of rhizobial genomes, the dual nature of their lifestyles, the protective and signaling roles of EPS, and the location of *pssG* and *pssI* in the operon seem to support such nature of these genes.

Interesting results concerning similar yet divergent roles of PssG and PssI in EPS subunit synthesis came from the complementation analyses of the mutants. The PssI protein overexpression in the Δ*pssI*(*pssI*) (with the gene delivered on mc-number vector), resembles a dominant negative effect where mutation of one enzyme in the multiprotein complex results in a reduction of the overall activity. This was confirmed by the bacterial two-hybrid screening results, where both PssG and PssI were shown to interact with other proteins involved in EPS synthesis. However, this feature was particularly evident for PssI, since this GT interacted in vivo with eight out of ten GTs involved in EPS biosynthesis in RtTA1; the exceptions were PssE and PssS. No such promiscuity was observed for PssG.

Why would one of the glucosyltransferases in a complex be so special? Exopolysaccharide synthesis is regulated at different levels of gene expression, with the prevalence of transcriptional regulation through DNA-binding regulatory proteins and sigma factors [51,52]. Post-translational regulation was also described, e.g., through the signaling cyclic di-GMP (c-di-GMP) molecule. However, in the latter case, the regulatory mechanism at the post-translational level was described only for homopolymeric polysaccharides synthesized and transported due to the activity of the processive glycosyltransferase called synthase [53]. Among several families of effector molecules of c-di-GMP, proteins containing the specific binding domain called PilZ—containing RXXXR and (D/N)X(S/A)XXG motifs—were described. When amino acid sequences of all 10 GTs involved in EPS synthesis in RtTA1 were inspected in search of the c-di-GMP binding motifs, we found their presence in 2 GTs: glucuronosyltransferase PssC (main chain synthesis) and glucosyltransferase PssI. Interestingly, both proteins are characterized by promiscuous interactions with other GTs in the bacterial two-hybrid screening. It will be interesting to find out in future experiments whether c-di-GMP is involved in the post-translational regulation of glycosyltransferase activity in the Wzx/Wzy-dependent synthesis system.

Both PssG and PssI proteins were shown to localize to the membrane, probably through the amphipathic α-helices localized in their C-termini. Both proteins are also more dissimilar in their C-parts, suggesting differences in the specificity/strength of UDP-hexose binding and recognition of the same oligosaccharide-lipid anchor. If this was the case and these enzymes indeed differed in the effectiveness in glucose transfer, it would be in good agreement with the varied level of the decrease in EPS synthesis in the single *pssI* and *pssG* mutants.

Counting the number of enzymatic steps involved in EPS subunit synthesis versus the number of GT-encoding genes made us ask the following question: are there too many genes or are they engaged in the synthesis in a manner more complex than ‘one gene—one activity’? Combining all the gathered data, the answer to this question seems to read: PssG and PssI proteins represent two, but not equivalent, components of the heterocomplex involved in the same step of glucose transfer to the EPS subunit side chain. However, PssI seems to be a major component of this complex: it is more independent in its membrane localization, is involved in more interactions within the EPS biosynthetic network, and is a good candidate for a protein regulated by post-translational c-di-GMP signaling.

## 4. Materials and Methods

### 4.1. Bacterial Strains and Culture Conditions

Bacterial strains used in this work are listed in Supplementary Table S3. *E. coli* strains were grown in lysogeny broth (LB) medium at 37 °C [54], and *R. leguminosarum* bv. *trifolii* strains were grown in TY (tryptone–yeast extract–calcium chloride) [54,55], M1 [56], or 79CA with 1% mannitol or glycerol at 28 °C [57]. The bacterial two-hybrid *E. coli* DHM1 strain was grown at 30 °C. Antibiotics were used at the following final concentrations: 100 μg/mL ampicillin, 40 μg/mL kanamycin, 5 (*E. coli*) or 10 μg/mL (*Rhizobium*) gentamicin, 10 μg/mL tetracycline, and 40 μg/mL rifampicin.

### 4.2. Bioinformatic Analyses

The putative operons were identified using Operon-mapper [58] and Rockhopper [59] tools. The input RNA-seq expression data for Rockhopper software were obtained previously (BioProject Accession: PRJNA894372, [34]). Promoter predictions in the sequence of the Pss-I region (GenBank Accession: MH595616, [26]) were performed using Neural Network Promoter Prediction [60] and PromoterHunter [61] tools, and Rho-independent terminators were searched for using the ARNold algorithm [62]. Simple pairwise comparison of nucleotide sequences of *pssG* and *pssI* were done with blastn, while the comparison of amino acid sequences of PssG and PssI using blastp [63]. For the alignment of PssG and PssI, Clustal Omega [64] was used and the result was visualized with Jalview [65]. Protein topology was predicted using the CCTOP [66]. Secondary structure prediction and protein homology modeling were performed with Phyre2 [67]. ConSurf and HeliQuest were used for prediction of amphipathic α-helices [68,69].

### 4.3. Total RNA Isolation and cDNA Synthesis

High-quality DNA-free total RNA was isolated from RtTA1 cells as described previously [34]. Briefly, RtTA1 cells were grown for 24 h in 79CA at 28 °C with shaking, then diluted to an OD_600_ of 0.05 in fresh 79CA medium and incubated until an OD_600_ of 0.7 was reached (≈10^9^ CFU). The cells were harvested by centrifugation at 4 °C and immediately submitted for RNA extraction with the GeneMATRIX Universal RNA Purification Kit (EURx Sp. z o.o., Gdańsk, Poland) according to the manufacturer’s protocol. Contaminating gDNA was removed with the TURBO DNA-free Kit (Thermo Fisher Scientific, Waltham, MA, USA), according to the rigorous DNase treatment. Three rounds of DNase (2 U) treatment for 30 min at 37 °C (6 U of DNase and 90 min of digestion in total) were performed. The quantity and quality of RNA was checked spectrophotometrically (Synergy H1 reader, Agilent Technologies, Inc., Santa Clara, CA, USA); fluorometrically (Qubit 2.0 Fluorometer with Qubit RNA High Sensitivity (HS) Assay Kit, Thermo Fisher Scientific, Waltham, MA, USA); in microcapillary electrophoresis (2100 Bioanalyzer Instrument with RNA 6000 Nano Kit, Agilent Technologies, Inc., Santa Clara, CA, USA); and in the PCR reaction. The isolated DNA-free total RNA was subjected to a reverse transcription reaction (0.5 µg RNA) with random primers according to the manufacturer’s recommendations (SuperScript IV VILO Master Mix with ezDNase Enzyme, Thermo Fisher Scientific, Waltham, MA, USA). The obtained cDNA was then used as a template in a series of PCR reactions (PCR Mix Plus, A&A Biotechnology, Gdańsk, Poland) with primers specific for pairs of adjacent genes of the RtTA1 Pss-I region (Supplementary Table S1).

### 4.4. DNA Techniques

Genomic and plasmid DNA isolations were performed with Bacterial & Yeast Genomic DNA Purification Kit (EURx Sp. z o.o., Gdańsk, Poland) and Plasmid Miniprep DNA Purification Kit (EURx Sp. z o.o., Gdańsk, Poland), respectively, according to the manufacturer’s protocols. Molecular cloning and transformation were performed according to standard protocols [70]. FastDigest restriction endonucleases were purchased in Thermo Fisher Scientific (Waltham, MA, USA). PCR was performed with high-fidelity Platinum SuperFi II DNA Polymerase (Thermo Fisher Scientific, Waltham, MA, USA) according to the manufacturer’s recommendations. The plasmids constructed and used in this work are listed in Supplementary Tables S2, S4, and S6–S8. The primers used in this work were synthesized at Genomed S. A. (Warsaw, Poland) and are listed in Supplementary Tables S1, S2, and S5–S7. Sanger DNA sequencing of plasmid constructs prepared in this work was performed in Genomed S. A. (Warsaw, Poland).

### 4.5. β-Galactosidase Activity Measurements of Transcriptional Fusions

Plasmids bearing *lacZ* transcriptional fusions resulted from the cloning of PCR products comprising predicted promoter regions into the respective restriction sites of the pMPK vector [71] (Supplementary Table S2). Obtained vectors were introduced into the RtTA1 cells via electrotransformation, as described by Garg et al. [72]. The RtTA1 strains carrying the *lacZ* transcriptional fusions in pMPK were grown overnight in 79CA medium in the presence of kanamycin. The cells were then washed twice with sterile water, diluted in fresh 79CA, TY, and M1 media, and grown to the mid-log phase. The level of *lacZ* expression was determined in Miller units, by assaying β-galactosidase activity with the ONPG (2-nitrophenyl-β-D-galactopyranoside, MP Biomedicals, LLC, Irvine, CA, USA) as a substrate, as described by Miller [73].

### 4.6. Construction of the ΔpssI and ΔpssG Single Mutants, ΔpssIΔpssG Double Mutant, ΔGT_10_ Mutant, and Their Complemented Derivatives

RtTA1 single gene mutants deleted for *pssI* or *pssG* were generated using the pCM351 allelic exchange vector [74], according to the procedure described previously [26,29]. The regions immediately flanking *pssI* or *pssG* were amplified by PCR using RtTA1 genomic DNA as a template. The purified 610 bp PCR product for the *pssI* upstream region was cloned into EcoRI–NdeI sites of pCM351 to produce pCGpssI-U. Subsequently, the purified 600 bp PCR product for the *pssI* downstream region was introduced between ApaI–SacI sites of pCGpssI-U, resulting in pCGpssI-UD. For the construction of the *pssG* gene mutagenesis vector, the purified 650 bp PCR product comprising the *pssG* upstream region was first cloned into KpnI–NdeI sites of pCM351 to produce pCGpssI-U, and then the purified 652 bp PCR product comprising the *pssG* downstream region was introduced between ApaI–SacI sites of pCGpssG-U, resulting in pCGpssI-UD. The plasmids pCGpssI-UD or pcGpssG-UD were then transferred to RtTA1 by biparental conjugation from *E. coli* S17-1 donor strain [75]. Gentamicin-resistant transconjugants obtained on TY medium containing rifampicin and gentamicin were subsequently screened for tetracycline sensitivity to identify potential *pssI* or *pssG* null mutants. The frequencies of the double-crossover events were 13.39% and 2.08% at the *pssG* or *pssI loci*, respectively. One such Δ*pssG*::Gm^R^, called Δ*pssG*(Gm^R^)—or Δ*pssI*::Gm^R^ mutant, called Δ*pssI*(Gm^R^)—was selected for further study. Analytical PCRs confirmed the successful allelic exchange. To remove the gentamicin resistance cassette, the plasmid pCM157 was introduced into Δ*pssG*(Gm^R^) or Δ*pssI*(Gm^R^) by electrotransformation. Tetracycline-resistant transformants were streaked for purity by two passages to obtain strains called Δ*pssG*[pCM157] or Δ*pssI*[pCM157], respectively, which produced only gentamicin-sensitive colonies. Next, pCM157 was cured from the obtained electrotransformants by five consecutive transfers on a nonselective medium to obtain the Δ*pssG* or Δ*pssI* mutant strains. Analytical PCRs were performed to confirm the successful deletion of the gentamicin resistance cassette. The sequencing of PCR-amplified product indicated expected recombination between *loxP* sites.

For the construction of the Δ*pssI*Δ*pssG* double mutant, the plasmid pCGpssG-UD was introduced into the Δ*pssI* single mutant by biparental mating. The frequency of the double-crossover event was 8.93%. To confirm the successful allelic exchange in such Δ*pssI*Δ*pssG*::Gm^R^, called Δ*pssI*Δ*pssG*, analytical PCR was performed.

To obtain the RtTA1 ΔGT_10_ mutant in which the *pssV*–*pssE* region and *pssA* gene were removed from the chromosome (which means that the mutant strain is devoid of all confirmed or putative genes encoding GTs responsible for EPS synthesis), a single mutant in the *pssV* gene was first constructed according to the method described above. The pCGpssV-UD vector was constructed by cloning the purified 675 bp PCR product comprising *pssV* upstream region into KpnI–NdeI sites of pCM351 to produce pCGpssV-U. The purified 563 bp PCR product comprising *pssV* downstream region into ApaI–BshTI sites of pCGpssV-U. pCGpssV-UD was then transferred to the wild-type RtTA1 by conjugation and Δ*pssV*::Gm^R^ null mutant, called Δ*pssV*(Gm^R^), was selected (double crossing-over rate was 11.97%). To remove the gentamicin resistance cassette, the plasmid pCM157 was introduced into Δ*pssV*(Gm^R^), resulting in Δ*pssV*[pCM157]. The Δ*pssV* single mutant strain was obtained after curing from pCM157. The ΔGT_9_ RtTA1 derivative was created using another plasmid for mutagenesis, called pCGpssE-UD. For this purpose, the purified 581 bp PCR product comprising *pssE* upstream region was cloned into KpnI–NdeI sites of pCM351 to produce pCGpssE-U, and then the purified 615 bp PCR product comprising *pssE* downstream region was cloned into ApaI–BshTI sites of pCGpssE-U. pCGpssE-UD was introduced into the Δ*pssV* by conjugation and Δ*pssV*Δ*pssE*::Gm^R^ double mutant, called Δ*pssV*Δ*pssE*, was selected (double crossing-over rate in *pssE locus* was 6.15%). After the introduction of plasmid pCM157, resulting in Δ*pssV*Δ*pssE*[pCM157] strain, Cre-mediated recombination, and curing of pCM157, the ΔGT_9_ mutant strain was generated. PCR reactions indicated expected recombination between *loxP* sites. The mutation at the *pssA locus* in the ΔGT_9_ strain was performed using the plasmid pCGpssA-UD constructed earlier [34]. The target strain with the Δ*pssA*::Gm^R^ mutation in the ΔGT_9_ mutant, named ΔGT_10_, was obtained with a double crossing-over frequency of 9.38%. The successful mutagenesis was confirmed by PCR and sequencing.

For the construction of the pBKpssI-C and pRKpssI-C plasmids used in complementation analyses, the 1199 bp PCR fragment comprising *pssI* was cloned between KpnI–XbaI sites of pBBR1-MCS2 or BamHI site of pRK7813, respectively. In the case of the pBKpssG-C plasmid, the 1065 bp PCR fragment comprising *pssG* gene was cloned between KpnI–SacI sites of pBBR1-MCS2. To construct a C-terminally His6-tagged version of PssI or PssG proteins, the 1066 bp fragment comprising the *pssI* gene or the 972 bp fragment comprising the *pssG* gene, both equipped with in-frame 3′-terminal His6-tag coding sequence, was cloned into XbaI–SacI or KpnI–BglII sites of pBBR1-MCS2, resulting in pBKpssI-His6 or pBKpssG-His6, respectively. The correctness of the constructed vectors was confirmed by sequencing. The obtained plasmids were transferred into the wild-type RtTA1 or the mutants through electrotransformation, resulting in WT(*pssI*-lc), WT(*pssI*-mc), Δ*pssI*(*pssI*-lc), Δ*pssI*(*pssI*-mc), Δ*pssI*(*pssI*his), Δ*pssG*(*pssG*), Δ*pssG*(*pssG*his), ΔGT_10_(*pssI*his), ΔGT_10_(*pssG*his), Δ*pssI*Δ*pssG*(*pssI*-lc), and Δ*pssI*Δ*pssG*(*pssG*) strains, respectively.

Bacterial strains, plasmids, and primers used and constructed during the mutagenesis procedure are listed in Supplementary Tables S3–S5, respectively.

### 4.7. General Analyses of Proteins

Proteins were routinely analyzed by SDS-PAGE and either visualized by PageBlue Protein Staining Solution (Thermo Fisher Scientific, Waltham, MA, USA) or electroblotted onto PVDF membrane (Immobilon-P, Merck KGaA, Darmstadt, Germany). Immunoblots were probed with the primary: anti-His_6_ antibodies (Roche, Basel, Switzerland), anti-PssP [76], or anti-PssB [77], and secondary anti-rabbit and anti-mouse IgG antibodies conjugated with alkaline phosphatase (Merck KgaA, Darmstadt, Germany). For S-tagged proteins, detection specific antibodies conjugated with alkaline phosphatase were used in a one-step procedure (Abcam PLC, Cambridge, United Kingdom).

### 4.8. Subcellular Localization Study

#### 4.8.1. Localization of Proteins in the Heterologous System

For expression of PssG and PssI proteins equipped with N-terminal His_6_-tag, the *pssG* and *pssI* genes were cloned into the pQE-30 vector (Supplementary Table S6). The pQE30*-his6-pssG/pssI* plasmid constructs were transformed into M15 (pREP4) chemically competent cells. In total, 10 mL bacterial cultures were grown in LB medium supplemented with antibiotics ampicillin and kanamycin. After the cell cultures reached an OD_600_ of 0.7, benzyl alcohol in a final concentration of 10 mM was added. After the induction with 0.1 mM isopropyl-β-D-galactopyranoside (IPTG, A&A Biotechnology, Gdańsk, Poland), expression was carried out at 21 °C for 18 h with shaking. The cells were harvested by centrifugation (5000 RCF, 4 °C, 5 min), and then resuspended in 2 mL of lysis buffer (50 mM NaH_2_PO_4_, 300 mM NaCl, pH 7.3) with lysozyme (1 mg/mL) (Merck KGaA, Darmstadt, Germany), protease inhibitor cocktail (10%) (Merck KGaA, Darmstadt, Germany), and viscolase (0.025 U/µL) (A&A Biotechnology, Gdańsk, Poland). The cell suspension was lysed with the FRENCH Pressure Cell Press (18,000 psi) (Thermo Fisher Scientific, Waltham, MA, USA) after 1 h incubation on ice. The resulting cell lysate was centrifuged to remove larger cell fragments (4000 RCF, 5 min, 4 °C) and then to remove the inclusion bodies (10,000 RCF, 10 min, 4 °C). The proteins were then further fractionated by centrifugation (100,000 RCF, 1 h, 4 °C) to obtain the membranes and soluble protein fractions. Subsequently, washing of the membranes with 1 M NaCl and the second round of centrifugation was performed to elute proteins associated with the membranes (peripheral proteins).

#### 4.8.2. Localization of Proteins in the Homologous System

The localization of the PssG and PssI proteins in the genetic background of *R. leguminosarum* was carried out in complemented versions of the Δ*pssI* and Δ*pssG* mutants, as well as in derivatives of ΔGT_10_ mutants complemented with the same plasmids (Supplementary Table S3). The strains were propagated in 20 mL of 79CA medium supplemented with kanamycin for 2 days. The cells were harvested by centrifugation (10,000 RCF, 15 min, 4 °C), next the obtained pellet was suspended in 2 mL of lysis buffer (50 mM NaH_2_PO_4_, 300 mM NaCl, pH 7.3) with lysozyme (1 mg/mL) (Merck KGaA, Darmstadt, Germany), protease inhibitor cocktail (10%) (Merck KGaA, Darmstadt, Germany), and viscolase (0.025 U/µL) (A&A Biotechnology, Gdańsk, Poland), and then incubated on ice for 1 h. After this time, disintegration was performed using the FRENCH Pressure Cell Press (18,000 psi) (Thermo Fisher Scientific, Waltham, MA, USA). The obtained clarified lysate was subjected to additional centrifugation at 10,000 RCF for 5 min at 4 °C. The lysate prepared in this way was fractionated according to the same procedure as the proteins after heterologous expression.

### 4.9. Purification and Solubilization of Inclusion Bodies

pCOLADuet-1 and pACYCDuet-1 vectors were used for cloning the *pssG* in front of the S-tag sequence, and *pssI* in front of the His_6_-tag, respectively (Supplementary Table S6). *E. coli* BL21(DE3) strain was transformed with the obtained pCOLAPssGSt and pACYCPssI plasmids. For effective overproduction of recombinant PssG and PssI, standard pre- and post-induction conditions were applied (37 °C before and after induction, 0.5 mM IPTG, A&A Biotechnology, Gdańsk, Poland). The *E. coli* cell pellet obtained from a 50 mL culture in LB was resuspended in 10 mL lysis buffer (55 mM NaH_2_PO_4_, 300 mM NaCl, pH 8.0) and supplemented with lysozyme (1 mg/mL) (Merck KGaA, Darmstadt, Germany) and protease inhibitors cocktail (0.5 mg/mL) (Merck KGaA, Darmstadt, Germany). After 1 h of agitation on ice, the cells were disintegrated using the FRENCH Pressure Cell Press (18,000 psi) (Thermo Fisher Scientific, Waltham, MA, USA). The samples were centrifuged for 30 min (5000 RCF, 4 °C) to remove cell debris, and then for 30 min (10,000 RCF, 4 °C) to separate the inclusion bodies from other cellular elements. The obtained pellet was suspended in an appropriate volume of washing buffer (5 mL of buffer per 1 g of the pellet) containing 4 M urea, 0.5 M NaCl, 1 mM EDTA, and 1 mg/mL DOC, and centrifuged (10,000 RCF, 15 °C, 15 min); this step was repeated twice. Solubilization of inclusion bodies was performed by resuspending the inclusion body pellet in solubilization buffer (2 mL of buffer per 1 g of the pellet) (6 M guanidine hydrochloride, 50 mM Tris-HCl, pH 8.0, 10 mM DTT) and incubation for 30 min at 30 °C, followed by centrifugation for 20 min (15,000 RCF, 21 °C).

### 4.10. Protein Refolding and Pull-Down Assay

Proteins dissolved in the solubilization buffer were suspended in the appropriate amount of refolding buffer (880 mM L-arginine, 55 mM Tris-HCl, 21 mM NaCl, 0.88 mM KCl, 100 mM EDTA, 200 mM GSH, 100 mM GSSG, pH 8.2) (Pierce Protein Refolding Kit, Thermo Fisher Scientific, Waltham, MA, USA) and left for a minimum of 12 h at 4 °C. After this time, the protein solution was incubated at 30 °C for 2 h. S-tag affinity chromatography resin was equilibrated by washing twice with the wash buffer (20 mM Tris-HCl, pH 7.5, 0.15 M NaCl, 0.1% (*v*/*v*) Triton X-100). Refolded proteins (100 µg each) were mixed together and applied to the resin. The flow-through fraction (F) was collected, then the resin was washed five times with wash buffer, and all fractions (W1–5) were collected. Proteins were then eluted twice with 3 M MgCl_2_ and fractions (E1–2) were collected (Figure S3). The remaining resin (Z) was also analyzed for the proteins remaining in it. Before loading proteins, all collected fractions were precipitated with acetone to avoid abnormal protein migration in the gel.

### 4.11. Topology Analyses

The *phoAlacZ*α reporter vector pPLE01 [39] was used to clone *pssG* and *pssI* genes and their fragments to create translational fusions with the examined gene at the 5′ end (Supplementary Table S7). For β-galactosidase and alkaline phosphatase activity screening, LB agar plates were supplemented with 80 μg/mL 5-bromo-4-chloro-3-indolyl phosphate (BCIP) (Roche, Basel, Switzerland) or 100 μg/mL 6-chloro-3-indolyl-β-D-galactoside (Red-Gal) (Glentham Life Sciences, Corsham, United Kingdom). Quantitation of β-galactosidase activity was performed as described in Marczak et al. [26] and quantification of alkaline phosphatase activity as described in [34].

### 4.12. Bacterial Two-Hybrid Analysis

Plasmids encoding fusions of glycosyltransferases and proteins involved in the transport and polymerization of EPS (PssT, PssP, PssL, and PssP2) were previously constructed [26,76,78] and are listed in Supplementary Table S8. Plasmids were co-transformed into *E. coli* DHM1 strain and interaction screening was performed using agar plates containing 40 μg/mL 5-bromo-4-chloro-3-indolyl-β-D-galactoside (X-Gal, A&A Biotechnology, Gdańsk, Poland), 0.5 mM IPTG (A&A Biotechnology, Gdańsk, Poland), ampicillin, and kanamycin. Quantitative measurement of β-galactosidase activity was performed in a plate format as described earlier [26].

### 4.13. Exopolysaccharide Analyses

Analyses of exopolysaccharides were performed as described in previous works [26,29]. Briefly, bacteria were cultured in 79CA with 0.5% glycerol, and exopolysaccharides were precipitated with 3 volumes of 95% ethanol from the cell-free supernatants of cultures grown with shaking for 3 days. The total sugar content was determined calorimetrically according to Dubois et al. [79] and calculated in glucose equivalents. The glycosyl composition of EPS was determined through GLC-MS of alditol acetates, according to Marczak et al. [78]. The molecular masses distribution in EPSs were determined by gel permeation chromatography on a column (1.0 cm × 90 cm) of Sepharose CL-6B (Merck KGaA, Darmstadt, Germany) using 1 M NaOH as eluent and a gravity flow at 0.2 mL/min. Fractions of 1 mL were collected. Blue Dextran (2 MDa) and Dextran T10 (10 kDa) were used as molecular weight standards.

### 4.14. Sensitivity Tests

SDS, DOC, ethanol, and NaCl-sensitivity assays were performed as described in Marczak et al. [26].

### 4.15. Statistical Analyses

The results were submitted for statistical analyses, which were performed with Statistica 13 software (StatSoft Polska, Kraków, Poland), using one-way analysis of variance (ANOVA) and the post hoc Tukey’s test.

## Supplementary Materials

The following supporting information can be downloaded at: https://www.mdpi.com/article/10.3390/ijms24044248/s1 [80,81,82,83].
