# Supplementary material for: Exopolysaccharide Biosynthesis in Rhizobium leguminosarum bv. trifolii Requires a Complementary Function of Two Homologous Glycosyltransferases PssG and PssI
Source: Int J Mol Sci. 2023 Feb 20;24(4):4248. doi: 10.3390/ijms24044248 (PMC9961541; doi:10.3390/ijms24044248)
Supplement: Supplementary file 1 [file ijms-24-04248-s001.zip › ijms-2144244-supplementary.pdf]

Supplementary Materials for

Exopolysaccharide biosynthesis in *Rhizobium leguminosarum* bv. *trifolii* requires a complementary function of two homologous glycosyltransferases PssG and PssI

Kamil Żebracki[1], Aleksandra Horbowicz[1], Małgorzata Marczak[1,*], Anna Turska-Szewczuk[1], Piotr Koper[1], Klaudia Wójcik[1], Marceli Romańczuk[1], Magdalena Wójcik[1], and Andrzej Mazur[1]

[1] Department of Genetics and Microbiology, Institute of Biological Sciences, Maria Curie-Skłodowska University, Akademicka 19 St., 20-033 Lublin, Poland; kamil.zebracki@mail.umcs.pl (K.Ż.); aleksandra9602@gmail.com (A.H.); malgorzata.marczak@mail.umcs.pl (M.M.); anna.turska-szewczuk@mail.umcs.pl (A.T.-S.); piotr.koper@mail.umcs.pl (P.K.); klaudiawojcik2398@gmail.com (K.W.); marcel.romanczuk11@gmail.com (M.R.), magdalena.wojcik2@mail.umcs.pl (M.W.); andrzej.mazur@mail.umcs.pl (A.M.)

***** Correspondence: malgorzata.marczak@mail.umcs.pl (M.M.)

This PDF file includes:
Supplementary Tables S1 to S8
Supplementary Figures S1 to S3
Supplementary References

Supplementary Table S1. List of primers used for RT-PCR of 5′-part of the Pss-I region

Amplicon (as in Fig. 1)	Amplified region	Primer	Sequence (5′–3′)	Reference
1	the last 416 bp of *mgl2*, *mgl2–regA* intergenic region, and the initial 427 bp of *regA*	mgl2-RT_Fw	CTCGGCCTACGACGACCCTTCA	This work
		regA-RT_Rv	ATAACCGAATCGTGCAACTCACTG	This work
2	the initial 40 bp of *mgl2*, *mgl2–pssV* intergenic region, and the initial 9 bp of *pssV*	mgl2-RT_Fw	TGGCGTGAACATAGGTGGCTG	This work
		pssV-RT_Rv	ATCGGGCAAGCTTGTCTTCTCC	This work
3	the last 85 bp of *pssV*, *pssV–pssW* intergenic region, and the initial 50 bp of *pssW*	pssV-RT_Fw	CGAGACCGACAGCCCACAA	This work
		pssW-RT_Rv	CATATCCGCTGCATCATACTCCA	This work
4	the last 593 bp of *pssW*, *pssW–pssS* intergenic region, and the initial 159 bp of *pssS*	pssW-RT_Fw	CAGCGGATATGCGGGATTGTGTT	This work
		pssS-RT_Rv	GTCATGAACCGGAACAGTGGATTT	This work
5	the last 453 bp of *pssS* and the initial 251 bp of *pssR*	pssS-RT_Fw	TTGAAGGAGGAAATCGAAGCACTG	This work
		pssR-RT_Rv	TGAAGGTAGAGCCCGCGTGTGAGG	This work
6	the last 10 bp of *pssS*, the whole *pssR*, and the initial 129 bp of *pssM*	pssR-RT_Fw	TCGGACTTGATGGTGCAGACAGGA	This work
		pssM-RT_Rv	GACTTTCGGCGGGTTGGTGTA	This work
7	the last 903 bp of *pssM*, *pssM–pssL* intergenic region, and the initial 372 bp of *pssL*	pssM-RT_Fw	ACTTTTTCTCGGCATCGGTTCG	This work
		pssL-RT_Rv	CAGCCGGGGCTCGTTGTAAAA	This work
8	the last 343 bp of *pssL*, *pssL–pssK* intergenic region, and the initial 398 bp of *pssK*	pssL-RT_Fw	GGGCCTGATTTCCACCGTAGTCAA	This work
		pssK-RT_Rv	TCGATGCGCTCAGGCGAACTGTAG	This work
9	the last 117 bp of *pssK*, *pssK–pssJ* intergenic region, and the initial 265 bp of *pssJ*	pssK-RT_Fw	GGCGGCACCGACCTTTCATA	This work
		pssJ-RT_Rv	CCCGGAAGGCATCAAGGAAGTCTT	This work
10	the last 401 bp of *pssJ*, *pssJ–pssI* intergenic region, and the initial 87 bp of *pssI*	pssJ-RT_Fw	TCTGGGACGATGGCGGTAGTTT	This work
		pssI-RT_Rv1	GTGCACCAAGGAATCCAAAGTATG	This work
11	the last 117 bp of *pssK*, *pssK–pssJ* intergenic region, the whole *pssJ*, *pssJ–pssI* intergenic region, and the initial 87 bp of *pssI*	pssK-RT_Fw	GGCGGCACCGACCTTTCATA	This work
		pssI-RT_Rv1	GTGCACCAAGGAATCCAAAGTATG	This work
12	the last 117 bp of *pssK*, *pssK–pssJ* intergenic region, the whole *pssJ*, *pssJ–pssI* intergenic region, and the initial 466 bp of *pssI*	pssK-RT_Fw	GGCGGCACCGACCTTTCATA	This work
		pssI-RT_Rv2	ACGGTCCGCTTTGGGTGTC	This work
13	the last 822 bp of *pssJ*, *pssJ–pssI* intergenic region, and the initial 466 bp of *pssI*	pssJ-RT_Fw2	CTCGGAAAGGGCAGCAAGGAAGA	This work
		pssI-RT_Rv2	ACGGTCCGCTTTGGGTGTC	This work
14	the last 285 bp of *pssI*, *pssI–pssH* intergenic region, and the initial 244 bp of *pssH*	pssI-RT_Fw	TGGATTCCGGCATCAAGTGTCA	This work
		pssH-RT_Rv	GGCGGAAGCCCAAAGTCAATG	This work
15	the last 774 bp of *pssH*, *pssH–pssG* intergenic region, and the initial 326 bp of *pssG*	pssH-RT_Fw	TTTGAGCCAAAACAAGGTAAGCAGTA	This work
		pssG-RT_Rv	CATGCAGCGATCGAACTCAACCA	This work
16	the last 113 bp of *pssG*, the whole *pssF*, and the initial 410 bp of *pssC*	pssG-RT_Fw	GGTTCTGGGCCATCTGGAAGTA	[1]
		pssC-RT_Rv	CGGTTTTTCTCCCGTGTCTCCAG	This work
17	the last 113 bp of *pssG* and the initial 168 bp of *pssF*	pssG-RT_Fw	GGTTCTGGGCCATCTGGAAGTA	[1]
		pssF-RT_Rv	CAGGATCCGCTCGCCATAGG	[1]
18	the last 264 bp of *pssF* and the initial 410 bp of *pssC*	pssF-RT_Fw	GGCACAAGCAGGGCAAAGACAC	This work
		pssC-RT_Rv	CGGTTTTTCTCCCGTGTCTCCAG	This work
19	the last 263 bp of *pssC*, *pssC–pssD* intergenic region, and the initial 129 bp of *pssD*	pssC-RT_Fw	GGAAATGGGTGCTGCAAAGACAAT	This work
		pssD-RT_Rv	AAGCAGCCCTGGGATGGTTGT	This work
20	the last 257 bp of *pssD* and the	pssD-RT_Fw	GCTTTTTCAGCGCCTTCTCCA	This work

	initial 255 bp of *pssE*	pssE-RT_Rv	AATGATCGGCTTCCCAAAACGC	This work
C	the initial 69 bp of *lasI*, *lasI–repA* intergenic region, and the initial 91 bp of *repAa*	lasI-RT_Fw	CCGGTACATTTGATCGAGAAGTGC	[1]
		repAa-RT-Rv	ACTGCAACTGCGCTGACAACATCT	[1]

The primer names include the annotation of adjacent genes. 'Fw' and 'Rv' refers to the forward and reverse primer, respectively.

Supplementary Table S2. List of plasmids and primers used for promoter activity assay

**Plasmid**	**Relevant description**[a]	**Reference**
pMPK	IncP, *mob*, promoterless *lacZ*, Tc^R, Km^R	[2]
PregA	pMPK with 296 bp KpnI-XbaI fragment comprising the last 40 bp of *mgl2*, *mgl2–regA* intergenic region, and the initial 81 bp of *regA*	This work
PpssV	pMPK with 675 bp KpnI fragment comprising the initial 40 bp of *mgl2* and *mgl2–pssV* intergenic region	This work
PpssW	pMPK with 743 bp KpnI-XbaI fragment comprising 693 bp upstream of *pssW* and the initial 50 bp of *regA*	This work
PpssI	pMPK with 524 bp KpnI-XbaI fragment comprising the last 307 bp of *pssJ*, *pssJ–pssI* intergenic region, and the initial 134 bp of *pssI*	This work
PpssH	pMPK with 650 bp KpnI-XbaI fragment comprising the last 332 bp of *pssI*, *pssI–pssH* intergenic region, and the initial 147 bp of *pssH*	This work
PpssG	pMPK with 501 bp KpnI-XbaI fragment comprising the last 322 bp of *pssH*, *pssH–pssG* intergenic region, and the initial 88 bp of *pssG*	This work
PpssD	pMPK with 604 bp KpnI-XbaI fragment comprising the last 263 bp of *pssC*, *pssC–pssD* intergenic region, and the initial 129 bp of *pssD*	This work
**Primer**	**Sequence (5′–3′)**[b]	**Reference**
PregAFwKpn	aaaggtaccATTGATTTCGGACGGCATTCGTCC	This work
PregARvXba	aatctagaGGTCATCCCGAGCGTCTTCC	This work
PpssVFwKpn	aaaggtaccTGGCGTGAACATAGGTGGCTG	This work
PpssVRvKpn	aaaggtaccGCTTGTCTTCTCCGGCGTGCGA	This work
PpssWFwKpn	aaaggtaccCTGCGCAACGGTTTCACAGGACT	This work
PpssWRvXba	aaatctagaACATACAACAGGGCGAGCGGAACC	This work
PpssIFwKpn	aaaggtaccGCCGACCCGCTTTGAGGATGC	This work
PpssIRvXba	aaatctagaTTGTTGTCGACGGCTATCAGTTCC	This work
PpssHFwKpn	aaaggtaccCCAATGGCGCCAAGTCCTATC	This work
PpssHRvXba	aaatctagaAGAGTCGTCGCTGCTGTTGTTG	This work
PpssGFwKpn	aaaggtaccCGGGGTTCGGCACATTGTCA	This work
PpssGRvXba	aaatctagaGCCCCAACATCGAATCCAGCATC	This work
PpssDFwKpn	aaaggtaccGGAAATGGGTGCTGCAAAGACAAT	This work
PpssDRvXba	aaatctagaAAGCAGCCCTGGGATGGTTGT	This work

[a] Abbreviations: Km^R, kanamycin resistance; Tc^R, tetracycline resistance

[b] Introduced restriction sites are underlined

Supplementary Table S3. List of *E. coli* and *R. leguminosarum* strains used in this work

Strain	Relevant description	Reference
E. coli		
DH5α	F^- φ80*lacZ*ΔM15 Δ(*lacZYA–argF*) U169 *deoR recA1 endA1 hsdR17* (r_k^-, m_k^+) *phoA supE44* λ^- *thi*-1 *gyrA96 relA1*, high efficiency transformation strain	[3]
S17-1	294 derivative, RP4-2-Tc::Mu-Km::Tn7 chromosomally integrated, mobilizing donor strain	[4]
M15 (pREP4)	host strain for the pQE-30 vector carries the pREP4 (*lacI*^{q} Km^r p14A *ori*) repressor plasmid	Qiagen
DHM1	Reporter strain for BTH system; F- *glnV*44(AS) *recA*1 *endA gyrA*96 *thi*-1 *hsdR*17 *spoT*1 *rfbD*1 *cya*-854	[5]
R. leguminosarum* bv. *trifolii		
RtTA1	wild-type strain, Str^R, Rif^R	[6]
Δ*pssI*(Gm^R)	RtTA1 Δ*pssI*::Gm^R	This work
Δ*pssI*[pCM157]	RtTA1 Δ*pssI* carrying pCM157 *cre* expressing vector	This work
Δ*pssI*	RtTA1 Δ*pssI*	This work
Δ*pssI*(*pssI*-lc)	RtTA1 Δ*pssI* carrying pRKpssI-C	This work
Δ*pssI*(*pssI*-mc)	RtTA1 Δ*pssI* carrying pBKpssI-C	This work
Δ*pssI*(*pssI*his)	RtTA1 Δ*pssI* carrying pBKpssI-His6	This work
WT(*pssI*-lc)	RtTA1 carrying pRKpssI-C	This work
WT(*pssI*-mc)	RtTA1 carrying pBKpssI-C	This work
Δ*pssG*(Gm^R)	RtTA1 Δ*pssG*::Gm^R	This work
Δ*pssG*[pCM157]	RtTA1 Δ*pssG* carrying pCM157 *cre* expressing vector	This work
Δ*pssG*	RtTA1 Δ*pssG*	This work
Δ*pssG*(*pssG*)	RtTA1 Δ*pssG* carrying pBKpssG-C	This work
Δ*pssG*(*pssG*his)	RtTA1 Δ*pssG* carrying pBKpssG-His6	This work
Δ*pssI*Δ*pssG*	RtTA1 Δ*pssI* strain carrying the second mutation Δ*pssG*::Gm^R	This work
Δ*pssI*Δ*pssG*(*pssG*)	RtTA1 Δ*pssI*Δ*pssG* double mutant carrying pBKpssG-C	This work
Δ*pssI*Δ*pssG*(*pssI*-lc)	RtTA1 Δ*pssI*Δ*pssG* double mutant carrying pRKpssI-C	This work
Δ*pssV*(Gm^R)	RtTA1 Δ*pssV*::Gm^R	This work
Δ*pssV*[pCM157]	RtTA1 Δ*pssV* carrying pCM157 *cre* expressing vector	This work
Δ*pssV*	RtTA1 Δ*pssV*	This work
Δ*pssV*Δ*pssE*	RtTA1 Δ*pssV* strain carrying the second mutation Δ*pssE*::Gm^R	This work
Δ*pssV*Δ*pssE*[pCM157]	RtTA1 Δ*pssV*Δ*pssE* double mutant carrying pCM157 *cre* expressing vector	This work
ΔGT_9	RtTA1 with deletion of the *pssV–pssE* region	This work
ΔGT_{10}	RtTA1 with deletion of the *pssV–pssE* region carrying additional *pssA*::Gm^R mutation	This work
ΔGT_{10}(*pssG*his)	ΔGT_{10} RtTA1 derivative carrying pBKpssG-His6	This work
ΔGT_{10}(*pssI*his)	ΔGT_{10} RtTA1 derivative carrying pBKpssI-His6	This work

Supplementary Table S4. List of plasmids used for mutagenesis and genetic complementation

Plasmid	Relevant characteristics	Reference
pCM351	*ori* ColE1, *oriT*, Ap^R, Gm^R, Tc^R, allelic exchange vector	[7]
pCM157	*ori* IncP, *oriT*, Tc^R, *cre* expression vector	[7]
pBBR1-MCS2	pBBR1 *rep*, *mob*, *lacZα* multi cloning site, Km^R, broad-host-range cloning vector	[8]
pRK7813	IncP, *oriT*, *cos*, *lacZα* multi cloning site, Tc^R, cosmid cloning vector	[9]
pCGpssI-U	pCM351 with 610 bp EcoRI–NdeI fragment comprising last 527 bp of *pssJ* and *pssJ–pssI* intergenic region	This work
pCGpssI-UD	pCGpssI-U with 600 bp ApaI–SacI fragment comprising *pssI–pssH* intergenic region and 429 bp of *pssH*	This work
pBKpssI-C	pBBR1MCS-2 with 1199 bp KpnI–XbaI fragment comprising *pssJ–pssI* intergenic region, *pssI*, and *pssI–pssH*	This work
pRKpssI-C	pRK7813 with 1199 bp BglII fragment comprising *pssJ–pssI* intergenic region, *pssI*, and *pssI–pssH*	This work
pBKpssI-C-His6	pBBR1MCS-2 with 1066 bp XbaI–SacI fragment comprising last 41 bp of *pssJ*, *pssJ–pssI* intergenic region, and *pssI* without stop codon, equipped with His_6-tag coding sequence and TAA stop codon	This work
pCGpssG-U	pCM351 with 650 bp KpnI–NdeI fragment comprising last 559 bp of *pssH* and *pssH–pssG* intergenic region	This wok
pCGpssG-UD	pCGpssG-U with 652 bp ApaI–SacI fragment comprising last 18 bp of *pssG* and 638 bp of *pssF*	This wok
pBKpssG-C	pBBR1MCS-2 with 1065 bp KpnI–SacI fragment comprising 90 bp upstream of *pssG* and *pssG*	This work
pBKpssG-C-His6	pBBR1MCS-2 with 972 bp KpnI–BglII fragment comprising *pssG* without stop codon, equipped with His_6-tag coding sequence and TGA stop codon	This work
pCGpssV-U	pCM351 with 675 bp KpnI–NotI fragment comprising 40 bp of *mgl2* and *mgl2–pssV* intergenic region	This work
pCGpssV-UD	pCGpssV-U with 563 bp ApaI–BshTI fragment comprising 563 bp downstream of *pssV*	This work
pCGpssE-U	pCM351 with 581 bp KpnI–NdeI fragment comprising 122 bp upstream of *pssD*, *pssD*, and 4 bp of *pssE*	This work
pCGpssE-UD	pCGpssE-U with 615 bp ApaI–BshTI fragment comprising last 16 bp of *pssE* and 599 bp upstream of *pssE*	This work
pCGpssA-UD	pCM351 with 584 bp KpnI–NotI fragment comprising last 7 bp of *pssB* and 577 bp of *pssB–pssA* intergenic region and 645 bp ApaI–SacI fragment comprising 645 bp downstream of *pssA*	[1]

Supplementary Table S5. List of primers used for construction of mutagenesis and complementation plasmids

Primer	Sequence (5′–3′)[a]	Reference	Application
pssI-U_FwEco	aagaattcTGCTGAATGCCACGGAAAGTCG	This work	amplification of genomic fragments for the construction of mutants obtained in this work
pssI-U_RvNde	aaacatatgCAGTTATCAACCCTCTTGGTGAAGTC	This work	
pssI-D_FwApa	aagggcccTCGATTGCATAGGAGGCAGTAATTT	This work	
pssI-D_RvSac	agagctcAAGAATCCCCAGATGCCCGTAAT	This work	
pssG-U_FwKpn	aaaggtaccCGGGCATCTGGGGATTCTTTA	This work	
pssG-U_RvNde	aaacatatgCAAACCCACTCGCCTCCTGAC	This work	
pssG-D_FwApa	aagggcccCAGGAGGTCGTGCATTGAAATTATCGGT	This work	
pssG-D_RvSac	agagctcTTTGCCCTGCTTGTGCCCGTGT	This work	
pssV-U_FwKpn	aaaggtaccTGGCGTGAACATAGGTGGCTG	This work	
pssV-U_RvNot	aagcggccgcGCTTGTCTTCTCCGGCGTGCGA	This work	
pssV-D_FwApa	aagggcccAGCCCCGTGCGTCCTTTCAG	This work	
pssV-D_RvBsh	aaaaccggtGCGCTCGTGATGGAAGATTGGT	This work	
pssE-U_FwKpn	aaaggtaccCAAAGCTTCGACCAACCAAACC	This work	
pssE-U_RvNde	aaacatatgTCAAAGGACAGCTCCTGCGTAGT	This work	
pssE-D_FwApa	aagggcccTATTGCCGCCGTCTGAACCC	This work	
pssE-D_RvBsh	aaaaccggtCCTTCGGAACATCCTTGACGG	This work	
pssI-C_FwKpn	aaaggtaccGCGCCGATCCCATTCGAACA	This work	amplification of genomic fragments for Δ*pssI* mutant complementation
pssI-C_RvXba	aatctagaGTGATGCTCCGGACCTCATTTCG	This work	
pssI-C_FwBgl	aaaagatctGCGCCGATCCCATTCGAACA	This work	
pssI-C_RvBgl	aaaagatctGTGATGCTCCGGACCTCATTTCG	This work	
pssI-C-His_FwXba	aatctagaGGCGCGAGTTTTTCGGTAAGA	This work	
pssI-C-His_RvSac	agagctcttaatgatgatgatgatggtgCTGCGTCATCGTCTGAGAAACGTATC	This work	
pssG-C_FwKpn	aaaggtaccGAAAGTACTAAACCGCGGCA	This work	amplification of genomic fragments for Δ*pssG* mutant complementation
pssG-C_RvSac	agagctcTCAATGCACGACCTCCTGCG	This work	
pssG-C-His_FwKpn	aaaggtaccctgacacaggaaacagctATGACGGATCCGAGAATTAGTGTC	This work	
pssG-C-His_RvBgl	aaaagatcttcaatgatgatgatgatggtgATGCACGACCTCCTGCGCTAGTC	This work	
pCMFw1	GGGTTCCGCGCACATTTC	[10]	validation of cloning and sequencing of the pCM351 derivatives
pCMRv1	GCTGCGTTCGGTCAAGGT	[10]	
pCMFw2	CCTAACAATTCGTTCAAGCCGA	[10]	
pCMRv2	CGCGCGAACGACATGGAG	[10]	
M13pUCf	CCCAGTCACGAAGTTGTAAAACG	Universal primer	validation of cloning and sequencing of the pBBR1-MCS2 and pRK7813 derivatives
M13pUCr	AGCGGATAACAATTTCACACAGG	Universal primer	

[a] Introduced restriction sites are underlined

Supplementary Table S6. List of plasmids and primers used for heterologous expression of *pssG* and *pssI* genes

**Plasmid**	**Relevant characteristics**	**Reference**
pQE-30	Expression vector, Ap^r	Qiagen
pQE30-*his6pssG*	The *pssG* gene cloned into the BglII–PstI site	This work
pQE30-*his6pssI*	The *pssI* gene cloned into the site BamHI–SmaI site	This work
pCOLADuet-1	Expression vector, Km^r	Novagen
pACYCDuet-1	Expression vector, Cm^r	Novagen
pCOLAPssGSt	pCOLADuet-1 vector with 988 bp BglII–KpnI fragment comprising *pssG* without stop codon, cloned into MCS-2	This work
pACYCPssI	pACYCDuet-1 vector with 963 bp SacI–NotI fragment comprising *pssI* without start codon, cloned into MCS-1	This work
**Primer**	**Sequence (5′–3′)[a]**	**Reference**
pssGpQE30/70fw	aaaagatctACGGATCCGAGAATTAGTGT	This work
pssGpQE30rv	aaactgcagTCAATGCACGACCTCCTGCG	This work
pssIpQE30/70fw	aaaagatctTCGGATCTCTTCGTCCAGCGT	This work
pssIpQE30rv	aaacccgggTTACTGCGTCATCGTCTGAG	This work
pQErevers	GTTCTGAGGTCATTACTGG	Qiagen
pQEpromoter	CCCGAAAAGTGCCACCTG	Qiagen
PssGDuetMcs2NdeIFw	aaacatatgACGGATCCGAGAATTAGTGTCATC	This work
PssGDuetMcs2KpnIRv	aaaggtaccATGCACGACCTCCTGCGC	This work
PssIDuetMcs1SacIFw	aaagagctcGTCGGATCTCTTCGTCAGCG	This work
PssIDuetMcs1NotIRv	aaagcggccgcTTACTGCGTCATCGTCTGAGAAAC	This work

[a] Introduced restriction sites are underlined

Supplementary Table S7. List of plasmids and primers used for topology mapping of PssG and PssI proteins

Plasmid	Relevant characteristics	Reference
pPLE01	pBluescript II SK(+) with *phoAlacZ*α from pMA632; Ap^r	[11]
pPLE01-G100	315 nt fragment spanning the 5′ end of the *pssG* gene cloned into the SacI-XbaI sites	This work
pPLE01-G173	543 nt fragment spanning the 5′ end of the *pssG* gene cloned into the SacI-XbaI sites	This work
pPLE01-G201	618 nt fragment spanning the 5′ end of the *pssG* gene cloned into the SacI-XbaI sites	This work
pPLE01-G324	987 nt fragment spanning the entire *pssG* gene cloned into the SacI-XbaI sites	This work
pPLE01-I148	459 nt fragment spanning the 5′ end of the *pssI* gene cloned into the SacI-XbaI sites	This work
pPLE01-I172	541 nt fragment spanning the 5′ end of the *pssI* gene cloned into the SacI-XbaI sites	This work
pPLE01-I269	822 nt fragment spanning the 5′ end of the *pssI* gene cloned into the SacI-XbaI sites	This work
pPLE01-I314	957 nt fragment spanning the entire *pssI* gene cloned into the SacI-XbaI sites	This work
pPLE01-T201	618 nt fragment spanning the 5′ end of the *pssT* gene cloned into the SacI-BamHI sites	[1]
pPLE01-T243	744 nt fragment spanning the 5′ end of the *pssT* gene cloned into the SacI-BamHI sites	[1]
**Primer**	**Sequence (5′–3′)[a]**	**Reference**
GFwrbspLEOSacI	aaagagctcGGAGAGTGGGTTTGATGAC	This work
GRv100AlapLEOXbaI	aaatctagaCGCATCTGCCTCGACGTC	This work
GRv173pLEOXbaI	aaatctagaCAATTCGCGACGCATGGTC	This work
GRv201ValpLEOXbaI	aaatctagaGACGATTTCCGTATCCTCT	This work
GRv324HispLEOXbaI	aaatctagaATGCACGACCTCCTGCGCTA	This work
IFwrbspLEOSacI	aaagagctcCAAGAGGGTTGATAACTG	This work
IRv148ValpLEOXbaI	aaatctagaGACTATCGCAAAGGTGGAT	This work
IRv172pLEOXbaI	aaatctagaCAACTCTCGCCGGACGAT	This work
IRv269AlapLEOXbaI	aaatctagaTGCGCTTTCGATCCAGGTC	This work
IRv314GlnpLEOXbaI	aaatctagaCTGCGTCATCGTCTGAGAAA	This work
pssTFWrbspLE01SacI	aaagagctcTCTAAGAGGTTGCAATGGCTTTG	[1]
pssTA201RVnew	aaaggatccCGCGGTCAGGCTGTCGA	[1]
pssTA243RVnew	aaaggatccCGCGTTTCGGCCCGGCT	[1]
phoAlacZseq	CATCCCATCGCCAATCAGCA	[1]

[a] Primers were designed in a way that ensured inclusion of a 15-nt-region in front of the ATG (TTG) codon, with the ribosome binding site (rbs). Letters G, I and T denote the *pssG, pssI* or *pssT* genes, respectively.

Supplementary Table S8. List of plasmids used for bacterial two-hybrid screening of PssG and PssI interactions

Plasmid	Relevant characteristics	Reference
pUT18	*cyaA*T18, Ap^R	[5]
pUT18C	*cyaA*T18, Ap^R	[5]
pKNT25	*cyaA*T25, Km^R	[5]
pKT25	*cyaA*T25, Km^R	[5]
pUT18C-zip	Two-hybrid control plasmid, Ap^R	[5]
pKT25-zip	Two-hybrid control plasmid, Km^R	[5]
pUT18-*pssADECSFGHIJ*	pUT18 with *pssADECSFGHIJ*	[12]
pUT18-*pssTL*	pUT18 with *pssTL*	[13]
pUT18-*pssP2*	pUT18 with *pssP2*	[14]
pUT18C-*pssADECSFGHIJ*	pUT18C with *pssADECSFGHIJ*	[12]
pUT18C-*pssPTL*	pUT18C with *pssPTL*	[13]
pUT18C-*pssP2*	pUT18C with *pssP2*	[14]
pKT25-*pssADECSFGHIJ*	pKT25 with *pssADECSFGHIJ*	[12]
pKT25-*pssPTL*	pKT25 with *pssPTL*	[13]
pKT25-*pssP2*	pKT25 with *pssP2*	[14]
pKNT25-*pssADECSFGHIJ*	pKNT25 with *pssADECSFGHIJ*	[12]

	T25-S	S-T25	T25-E	E-T25	T25-D	D-T25	T25-A	A-T25	T25-C	C-T25	T25-F	F-T25	T25-I	I-T25	T25-G	G-T25	T25-J	J-T25	T25-H	H-T25	T25-P	T25-T	T25-L	T25-P2
S-T18													92.4	99.9	100.1	99.3								
T18-S													95.6	108.7	105.6	100.1								
E-T18													95.0	110.6	100.7	90.6								
T18-E													95.7	107.7	102.1	99.1								
D-T18													108.3	107.2	102.3	102.1								
T18-D													103.2	118.2	110.1	103.2								
A-T18													104.5	99.7	117.5	96.7								
T18-A													241.2	94.0	117.8	103.2								
C-T18													237.1	212.3	113.4	128.8								
T18-C													392.7	99.7	91.4	387.4								
F-T18													97.4	90.3	98.1	97.0								
T18-F													488.5	94.3	107.6	101.5								
I-T18	94.9	100.9	87.7	109.0	179.2	156.9	623.5	114.8	786.1	354.9	432.5	441.2	854.5	236.5	104.7	725.2	440.5	384.0	100.4	91.8	136.9	282.9	105.2	405.2
T18-I	92.9	99.9	89.9	99.7	99.7	99.7	555.5	96.8	533.9	807.7	101.4	286.0	547.1	452.4	119.7	218.7	312.1	211.7	101.5	99.8	105.4	106.8	108.4	141.3
G-T18	108.0	100.1	95.1	98.3	95.0	410.0	134.5	100.3	121.9	119.7	109.8	103.0	533.5	98.1	103.9	353.7	180.3	150.9	93.8	102.9	108.8	112.7	101.3	136.7
T18-G	97.8	89.1	100.2	117.9	92.5	109.8	105.8	100.4	170.7	103.7	92.4	97.2	366.7	92.9	116.6	93.6	107.8	105.7	98.3	97.0	104.3	103.9	104.3	105.6
J-T18													130.2	296.3	83.6	107.4								
T18-J													503.5	91.6	732.7	100.2								
H-T18													406.1	89.2	94.4	97.0								
T18-H													124.9	89.9	89.8	89.6								
T18-P													122.5	119.2	107.0	114.9								
T-T18													120.4	111.5	114.0	118.6								
T18-T													114.0	108.8	119.2	112.0								
L-T18													105.9	106.7	109.9	118.4								
T18-L													101.3	111.7	118.7	111.9								
P2-T18													111.1	114.2	107.2	111.2								
T18-P2													122.1	111.1	111.6	123.6								

	T25-S	S-T25	T25-E	E-T25	T25-D	D-T25	T25-A	A-T25	T25-C	C-T25	T25-F	F-T25	T25-I	I-T25	T25-G	G-T25	T25-J	J-T25	T25-H	H-T25	T25-P	T25-T	T25-L	T25-P2
S-T18													9.5	3.0	2.4	7.0								
T18-S													3.0	3.9	1.9	4.0								
E-T18													2.0	6.4	8.6	3.3								
T18-E													4.2	4.3	4.9	3.6								
D-T18													2.2	9.4	7.4	20.6								
T18-D													3.3	0.8	9.6	12.3								
A-T18													3.5	2.2	10.1	5.4								
T18-A													12.6	3.1	7.2	5.2								
C-T18													23.4	13.1	13.5	11.1								
T18-C													36.8	4.4	22.2	21.8								
F-T18													5.0	3.6	2.5	4.4								
T18-F													12.8	3.2	7.5	7.4								
I-T18	7.0	7.8	12.9	0.9	15.7	49.2	29.6	11.6	8.7	25.2	12.0	48.9	16.3	14.3	13.9	35.5	51.1	93.3	9.1	6.2	2.7	5.3	1.6	14.4
T18-I	7.4	9.7	9.6	14.1	10.4	1.4	22.1	9.7	49.6	20.8	11.8	10.6	11.0	47.4	6.2	17.5	26.3	105.9	8.1	2.6	0.7	1.5	0.7	26.0
G-T18	12.8	11.5	12.6	11.7	12.3	24.3	3.8	2.9	4.1	2.2	3.7	8.7	21.6	4.9	4.4	18.4	12.1	18.9	3.3	3.3	3.5	2.5	3.1	1.6
T18-G	12.5	2.2	6.3	4.7	15.7	6.5	1.7	4.5	6.0	3.6	9.3	7.8	52.2	3.7	13.8	7.4	5.8	9.9	2.9	4.1	1.6	1.3	1.1	0.8
J-T18													11.2	38.6	11.0	5.9								
T18-J													43.0	3.5	42.7	7.1								
H-T18													38.6	4.0	1.0	9.2								
T18-H													13.7	5.1	6.6	7.8								
T18-P													0.5	1.5	12.1	0.7								
T-T18													1.0	1.3	1.5	2.2								
T18-T													1.5	10.4	3.5	1.5								
L-T18													3.4	5.1	1.1	1.2								
T18-L													6.0	16.3	1.4	8.8								
P2-T18													21.4	4.5	8.7	9.9								
T18-P2													22.7	9.1	1.0	12.6								

Supplementary Figure S1. The activity of β-galactosidase measured in *E. coli* DHM1 strain carrying pairs of fusion plasmids. Letters A, D, E, etc. in plasmid names stand for the last letter in the name of the GT gene, and the position of the letter indicates N- or C-terminal localization of the glycosyltransferase. The top panel presents mean activity (Miller units), the bottom panel – standard deviations. Blue boxes shade significant activities. Negative controls [pUT18(pUT18C) × pKT25(pKNT25)] gave 90.7 ± 4.9 – 98.9 ± 2.1 Miller units. Positive control (pUT18zip × pKT25zip) gave 727.1 ± 43.0. Pink surface – GTs, grey surface – polymerization and transport proteins.

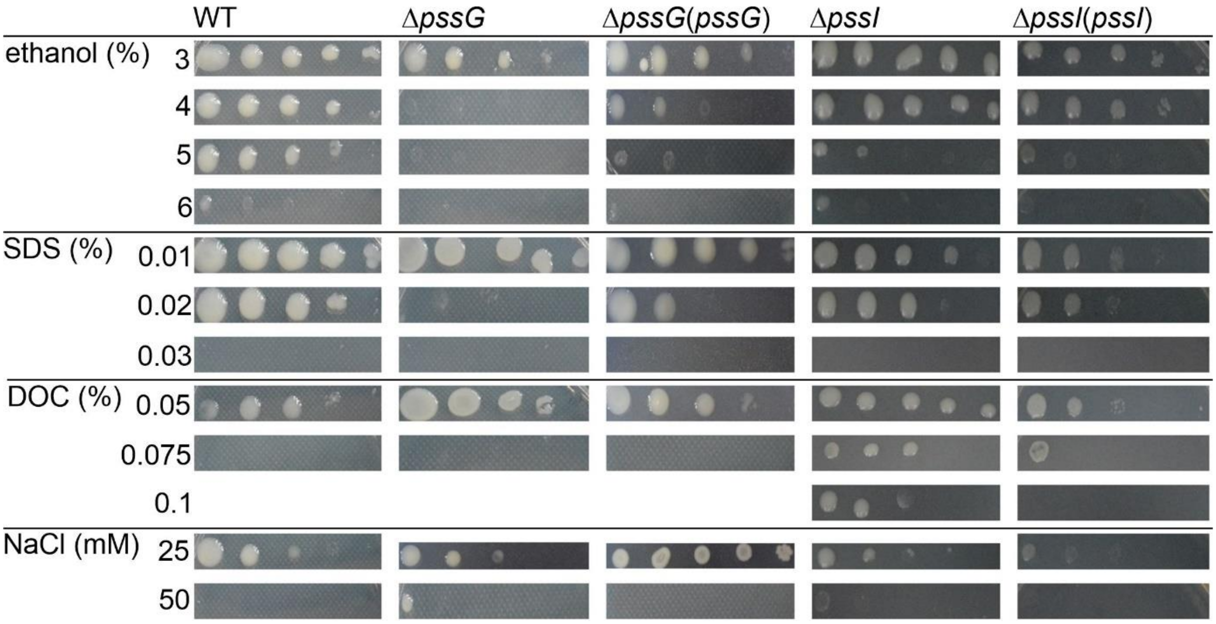

Supplementary Figure S2. Plate sensitivity tests. *pssG* deletion is associated with increased sensitivity to ethanol and SDS, while *pssI* deletion – a slight decrease in sensitivity to excess DOC.

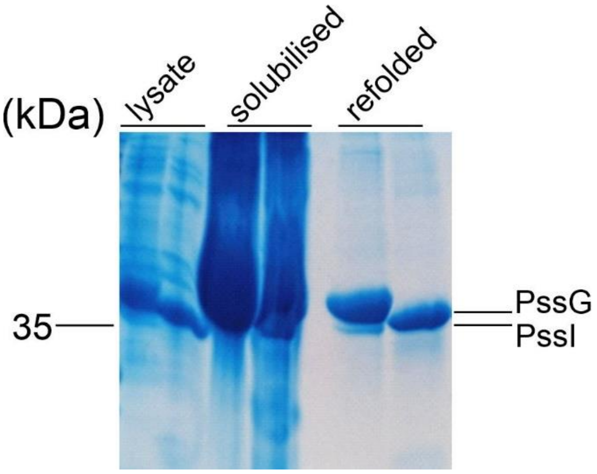

Supplementary Figure S3. Recombinant PssG-Stag and PssI-His6 proteins at different stages of expression, purification, solubilization, and refolding. SDS-PAGE was performed for samples of clarified lysate before inclusion bodies removal (left), proteins after solubilization of pure inclusion bodies (middle), and recombinant PssG and PssI after refolding (right) (in each pair of lanes left represents PssG and right – PssI, respectively).

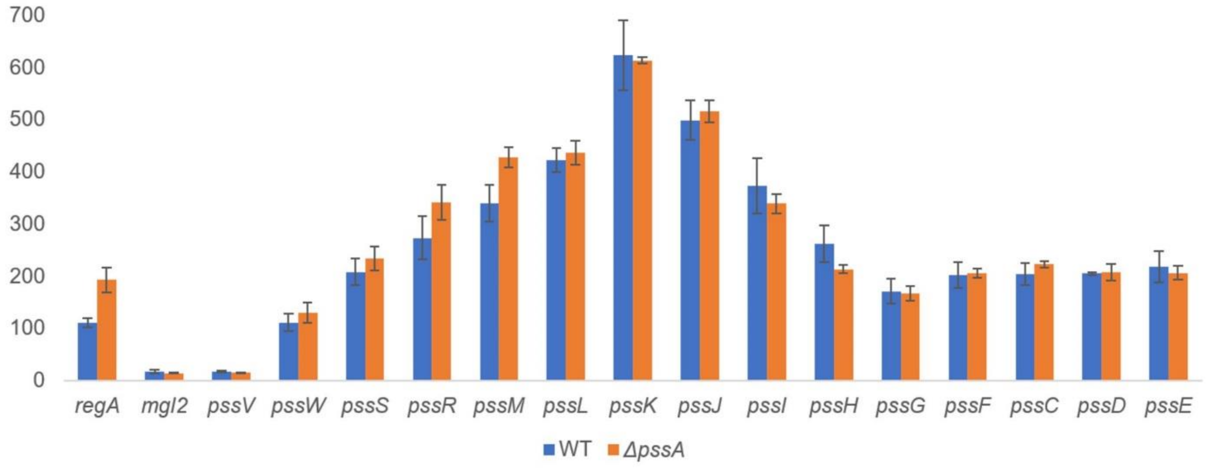

Supplementary Figure S4. Level of gene expression (transcripts per million, TPM ± SD) of the 5′ end of the Pss-I region in RtTA1 wild-type (WT, blue bars) and Δ*pssA* mutant (orange bars) strains. Transcriptomic data has been obtained and published previously (BioProject Accession: PRJNA894372; [1])

Supplementary References

1. Marczak, M.; Żebracki, K.; Koper, P.; Horbowicz, A.; Wójcik, M.; Mazur, A., A new face of the old gene: deletion of the *pssA*, encoding monotopic inner membrane phosphoglycosyl transferase in *Rhizobium leguminosarum*, leads to diverse phenotypes that could be attributable to downstream effects of the lack of exopolysaccharide. Article under review at *International Journal of Molecular Sciences*
2. Żebracki, K.; Koper, P.; Marczak, M.; Skorupska, A.; Mazur, A. Plasmid-Encoded RepA Proteins Specifically Autorepress Individual *repABC* Operons in the Multipartite *Rhizobium leguminosarum* bv. *trifolii* Genome. *PloS One* **2015**, 10, e0131907, doi:10.1371/journal.pone.0131907.
3. Sambrook, J.; Russell, D.W., *Molecular cloning: a laboratory manual*, 3rd ed.; Cold Spring Harbor Laboratory Press: Cold Spring Harbor, New York, 2001.
4. Simon, R.; Priefer, U.; Pühler, A. A broad host range mobilization system for *in vivo* genetic engineering: transposon mutagenesis in Gram negative bacteria. *Bio/Technology* **1983**, 1, 784-791, doi:10.1038/nbt1183-784.
5. Karimova, G.; Pidoux, J.; Ullmann, A.; Ladant, D. A bacterial two-hybrid system based on a reconstituted signal transduction pathway. *Proc. Natl. Acad. Sci. USA* **1998**, 95, 5752-5756, doi:10.1073/pnas.95.10.5752.
6. Chakravorty, A.K.; Zurkowski, W.; Shine, J.; Rolfe, B.G. Symbiotic nitrogen fixation: molecular cloning of *Rhizobium* genes involved in exopolysaccharide synthesis and effective nodulation. *J. Mol. Appl. Genet.* **1982**, 1, 585-596.
7. Marx, C.J.; Lidstrom, M.E. Broad-host-range *cre-lox* system for antibiotic marker recycling in gram-negative bacteria. *BioTechniques* **2002**, 33, 1062-1067, doi:10.2144/02335rr01.
8. Kovach, M.E.; Elzer, P.H.; Hill, D.S.; Robertson, G.T.; Farris, M.A.; Roop, R.M., 2nd; Peterson, K.M. Four new derivatives of the broad-host-range cloning vector pBBR1MCS, carrying different antibiotic-resistance cassettes. *Gene* **1995**, 166, 175-176, doi:10.1016/0378-1119(95)00584-1.
9. Jones, J.D.; Gutterson, N. An efficient mobilizable cosmid vector, pRK7813, and its use in a rapid method for marker exchange in *Pseudomonas fluorescens* strain HV37a. *Gene* **1987**, 61, 299-306, doi:10.1016/0378-1119(87)90193-4.
10. Marczak, M.; Żebracki, K.; Koper, P.; Turska-Szewczuk, A.; Mazur, A.; Wydrych, J.; Wójcik, M.; Skorupska, A. Mgl2 Is a hypothetical methyltransferase involved in exopolysaccharide production, biofilm formation, and motility in *Rhizobium leguminosarum* bv. *trifolii*. *Mol. Plant Microbe Interact.* **2019**, 32, 899-911, doi:10.1094/MPMI-01-19-0026-R.
11. Islam, S.T.; Taylor, V.L.; Qi, M.; Lam, J.S. Membrane topology mapping of the O-antigen flippase (Wzx), polymerase (Wzy), and ligase (WaaL) from *Pseudomonas aeruginosa* PAO1 reveals novel domain architectures. *mBio* **2010**, 1, doi:10.1128/mBio.00189-10.
12. Marczak, M.; Wójcik, M.; Żebracki, K.; Turska-Szewczuk, A.; Talarek, K.; Nowak, D.; Wawiórka, L.; Sieńczyk, M.; Łupicka-Słowik, A.; Bobrek, K.; Romańczuk, M.; Koper, P.; Mazur, A. PssJ Is a Terminal Galactosyltransferase Involved in the Assembly of the Exopolysaccharide Subunit in *Rhizobium Leguminosarum* bv. *Trifolii*. *Int. J. Mol. Sci.* **2020**, 21, doi:10.3390/ijms21207764.
13. Marczak, M.; Dźwierzyńska, M.; Skorupska, A. Homo- and heterotypic interactions between Pss proteins involved in the exopolysaccharide transport system in *Rhizobium leguminosarum* bv. *trifolii*. *Biol. Chem.* **2013**, 394, 541-559, doi:10.1515/hsz-2012-0161.
14. Marczak, M.; Matysiak, P.; Kutkowska, J.; Skorupska, A. PssP2 is a polysaccharide co-polymerase involved in exopolysaccharide chain-length determination in *Rhizobium leguminosarum*. *PloS One* **2014**, 9, e109106, doi:10.1371/journal.pone.0109106.
